# Detection of transcriptome-wide microRNA–target interactions in single cells with agoTRIBE

**DOI:** 10.1038/s41587-023-01951-0

**Published:** 2023-09-21

**Authors:** Vaishnovi Sekar, Emilio Mármol-Sánchez, Panagiotis Kalogeropoulos, Laura Stanicek, Eduardo A. Sagredo, Albin Widmark, Evangelos Doukoumopoulos, Franziska Bonath, Inna Biryukova, Marc R. Friedländer

**Affiliations:** 1 Science for Life Laboratory, Department of Molecular Biosciences, The Wenner-Gren Institute, Stockholm University, Stockholm, Sweden; 2 Centre for Palaeogenetics, Stockholm, Sweden; 3 Department of Molecular Biosciences, The Wenner-Gren Institute, Stockholm University, Stockholm, Sweden; 4 Department of Medicine, Karolinska Institutet, Solna, Sweden

**Keywords:** Functional genomics, miRNAs, RNA sequencing

## Abstract

MicroRNAs (miRNAs) exert their gene regulatory effects on numerous biological processes based on their selection of target transcripts. Current experimental methods available to identify miRNA targets are laborious and require millions of cells. Here we have overcome these limitations by fusing the miRNA effector protein Argonaute2 to the RNA editing domain of ADAR2, allowing the detection of miRNA targets transcriptome-wide in single cells. miRNAs guide the fusion protein to their natural target transcripts, causing them to undergo A>I editing, which can be detected by sensitive single-cell RNA sequencing. We show that agoTRIBE identifies functional miRNA targets, which are supported by evolutionary sequence conservation. In one application of the method we study microRNA interactions in single cells and identify substantial differential targeting across the cell cycle. AgoTRIBE also provides transcriptome-wide measurements of RNA abundance and allows the deconvolution of miRNA targeting in complex tissues at the single-cell level.

## Abstract (web summary)

Functional miRNA targets are identified transcriptome-wide in single cells.

## Main

miRNAs are small noncoding RNAs that posttranscriptionally regulate the expression of protein-coding genes^1^. Mechanistically they guide Argonaute effector proteins to messenger RNA targets, allowing Argonaute and cofactors to inhibit translation and/or promote degradation of target mRNAs^2–5^. miRNAs are found in virtually all multicellular animals and plants and play important roles in numerous biological processes, including development, formation of cell identity and human diseases such as cancer^1,6–8^. The human genome harbors hundreds of distinct miRNA genes^9^, each of which can putatively regulate hundreds of target genes. The function of each individual miRNA is defined by its specific target repertoire and thus, to understand the function of a given miRNA, it is necessary to map its targets. The current state-of-the-art method to do so is crosslinking-immunoprecipitation-sequencing (CLIP-seq), which applies ultraviolet light to crosslink the Argonaute protein to its mRNA targets in cells, then isolates the protein using antibodies and uses next-generation sequencing to profile the bound RNA targets^10,11^. This method has brought many new insights to the miRNA field yet it has some inherent limitations. First, because isolation with antibodies is inefficient, it requires as input in the order of millions of cells, making it unsuited for samples with limited material—not to mention in single cells. By averaging over millions of cells, CLIP-seq masks potential cell-to-cell variation. Second, the method is laborious and requires many specialized protocol steps, including ultraviolet crosslinking and immunoprecipitation.

We here present our method agoTRIBE, which circumvents these limitations. We show that our method yields results that are consistent with the more laborious CLIP-seq method. The reported miRNA targets are supported by evolutionary sequence conservation and are subject to functional miRNA repression. In addition, we show that agoTRIBE can be applied to the detection of miRNA–target interactions in human single cells, and to deconvolution of miRNA targeting in distinct phases of the cell cycle without the need for physical cell sorting.

## Results

**Fig. 1:**
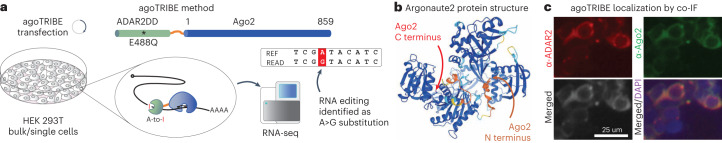
agoTRIBE fuses AGO2 and the editing domain of ADAR2. **a**, Schematic representation of the agoTRIBE method. The agoTRIBE approach fuses human Argonaute2 with the adenosine deaminase domain of human ADAR2, carrying a hyperactive mutation (E488Q) and depositing edits on the targeted transcripts. The edited nucleotides can be detected as A>G substitutions by either standard or single-cell RNA-seq. Top: schematic representation of the N-terminal tagging of human Argonaute2. **b**, Human Argonaute2 protein structure prediction using AlphaFold2. Tagging of the C terminus, which is embedded in the protein structure, could result in a misfolded protein unable to load miRNAs. **c**, Immunofluorescence staining to visualize agoTRIBE. AGO2 (green) and ADAR2 (red) immunofluorescence costaining (co-IF) were used to detect agoTRIBE, while DAPI (magenta) was used for nuclear staining. Each microscopy experiment was performed in at least two replicates and a representative experiment is shown. Colocalization in cytoplasmic foci suggests that agoTRIBE is present in P-bodies.

To develop our method we leveraged on the TRIBE approach^12^, in which an RNA-binding protein of interest (in our case, Argonaute2) is fused to the RNA editing domain of ADAR2. The RNA-binding protein part leads the fusion protein to its natural targets while the editing domain deaminates adenosines to inosines (A>I) in the RNA target, in effect leaving nucleotide conversions that can be detected by sequencing as A>G substitutions (Fig. 1). These substitutions can in principle be detected by single-cell RNA sequencing (RNA-seq), and the method avoids lossy isolation because it does not use immunoprecipitation. To tailor agoTRIBE for Argonaute proteins we made three modifications to the original TRIBE approach: (1) we used a hyperactive version of the ADAR2 deaminase domain, in which a E488Q substitution results in increased editing^13,14^; (2) we connected the Argonaute2 and ADAR2 domains with a 55-amino-acids-long flexible linker; and (3) we fused the ADAR2 domain to the N terminus of Argonaute2, because the protein structure of Argonaute2 indicates that fusion to the C terminus would be detrimental to the loading of guide miRNAs^15–19^ (Fig. 1b). We confirmed that tagging Argonaute2 with the ADAR2 editing domain does not change its cytoplasmic localization (Fig. 1c), nor its colocalization with TNRC6B, a P-body marker (Supplementary Fig. 1a). In particular, we found that agoTRIBE partly locates to cytoplasmic foci that are similar to P-bodies, which are known to be interconnected with miRNA function^20–22^ (Fig. 1c).

**Fig. 2:**
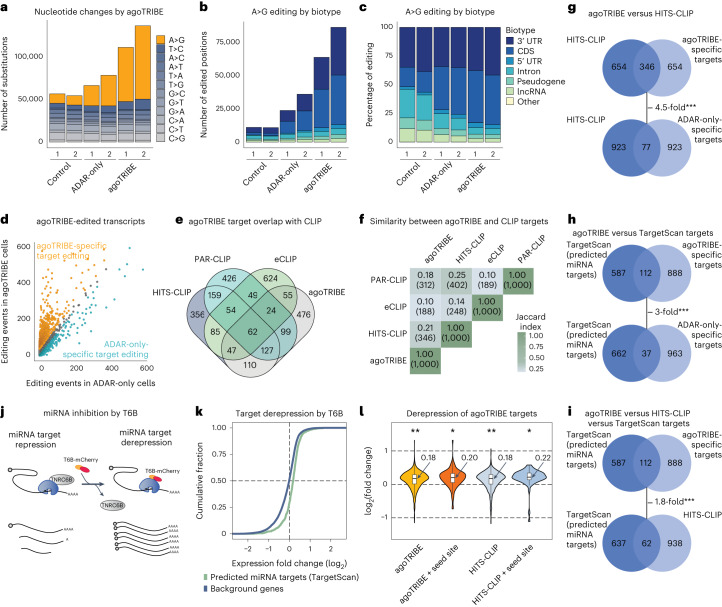
agoTRIBE detects miRNA targets through RNA editing. **a**, A>G editing (colored orange), indicative of ADAR2 editing, specifically increased in agoTRIBE-transfected cells compared with both control cells transfected with eGPF-Ago2 and cells transfected with the ADAR2 editing domain without Argonaute2 (ADAR-only controls). **b**, Editing increased specifically in 3′ UTR and coding sequences (CDS) and remained constant in other transcript types. **c**, As in **b**, but represented as percentages of editing. **d**, Editing in agoTRIBE-transfected versus ADAR-only-transfected cells. Each dot corresponds to one gene. Genes colored orange have substantially more editing in agoTRIBE cells while those colored light blue have substantially more editing in ADAR-only cells (Methods). Genes colored gray have comparable editing in both conditions. **e**, Venn diagram of top 1,000 miRNA targets reported individually by agoTRIBE, HITS-CLIP, PAR-CLIP and eCLIP. **f**, Jaccard similarity index values of top target sets reported by each of the four methods. **g**–**i**, Venn diagrams of overlaps between agoTRIBE and HITS-CLIP targets (**g**); agoTRIBE and TargetScan targets (**h**); and agoTRIBE, HITS-CLIP and TargetScan targets (**i**). **j**, Schematic representation of global miRNA inhibition by T6B. **k**, Derepression of miRNA targets predicted by TargetScan following T6B-mCherry transfection. In total, 609 TargetScan targets and 14,515 background transcripts were profiled. **l**, Increase in expression of agoTRIBE and HITS-CLIP targets following T6B transfection. ‘Seed site’ indicates that the transcript harbors a conserved binding site for one of the ten most abundant miRNAs in HEK-293T cells. Boxes indicate median and 25th and 75th quantiles, and whiskers indicate lowest and largest values. Significance was calculated using the Wasserstein distance (Methods); *P* values, left to right: 0.00025, 0.021, 0.00025, 0.012. *P < 0.05; **P < 0.001. In total, 1,000 agoTRIBE targets (112 with seed sites) and 996 HITS-CLIP targets (62 with seed sites) were profiled.

When we transiently expressed agoTRIBE in ~50,000 human HEK-293T cells (Methods), we observed that A>G nucleotide substitutions—as expected by ADAR2-mediated editing—increased substantially compared with control cells where editing is low, consistent with ADAR2 being undetectable in this cell type (Fig. 2a, Supplementary Tables 1–4 and Supplementary Fig. 1b–d). In contrast, cells transfected with only the ADAR2 deaminase domain without Argonaute2—henceforward referred as ‘ADAR-only’—increased only moderately the number of A>G substitutions, suggesting the importance of miRNA guidance for newly detected editing (Fig. 2a). Of note, other types of nucleotide substitution remained largely unchanged among the analyzed conditions, indicating specific ADAR2-mediated editing. In addition, we observed that editing in mRNA exonic regions specifically increased while editing in intronic regions and noncoding transcripts such as long noncoding RNAs and pseudogenes remained constant (Fig. 2b,c). This is consistent with miRNAs targeting mature mRNAs in the cytoplasm, while there is little evidence of miRNAs targeting nontranslating sequences such as introns or lncRNA transcripts^23^, which are most commonly located in the nucleus. We note that the agoTRIBE construct did not appear to substantially change the miRNA profile of transfected cells (Supplementary Fig. 2). In summary, we observed highly increased editing in mRNA transcripts that likely correspond to natural miRNA targets.

We next compared agoTRIBE with state-of-the-art CLIP-seq methods. First, to discern miRNA-guided editing from background editing—including that of endogenously expressed ADAR2—we compared editing patterns following agoTRIBE transfection relative to ADAR-only E488Q controls (Fig. 2d). We assumed that transcript editing specific to agoTRIBE is guided by miRNAs while editing specific to ADAR-only represents editing background activity. To do so, we compared the total number of editing events per gene in the two conditions—agoTRIBE versus ADAR-only—and focused on the top 1,000 putative mRNA targets showing specifically increased editing following agoTRIBE transfection (Fig. 2d). We then compared our list with the top 1,000 targets identified by different variations of the Argonaute CLIP-seq methodology^24–26^. We found that the agoTRIBE top 1,000 targets have substantial overlap with CLIP-seq targets and do not represent an outlier group with an excess of unique targets not found by CLIP-seq methods (Fig. 2e). In fact, the enhanced crosslinking and immunoprecipitation (eCLIP) method reports more targets (624 unique targets) not shared with any other method than does agoTRIBE (476 unique targets; Fig. 2e). Comparing the similarity between the target sets reported by each of the four methods using the Jaccard index, we found that high-throughput sequencing of RNA isolated by crosslinking and immunoprecipitation (HITS-CLIP) and photoactivatable ribonucleoside-enhanced crosslinking and immunoprecipitation (PAR-CLIP) resemble each other the most (Jaccard index = 0.25; Fig. 2f), while agoTRIBE also has strong similarity to both of these methods (Jaccard index = 0.21 and 0.18, respectively), eCLIP in contrast had less resemblance to the other two CLIP methods (Jaccard index = 0.14 and 0.10, respectively). More than half of the targets (525) reported by agoTRIBE were supported by one or more CLIP-seq methods (Fig. 2e). We considered that the overlap between methods could be due to undetected biases—for instance, that highly expressed transcripts might be more efficiently detected by both CLIP-seq and agoTRIBE. We therefore compared the overlap between the top 1,000 targets from agoTRIBE and HITS-CLIP with that between our ADAR-only controls and HITS-CLIP (Fig. 2g). We found that the overlap between agoTRIBE and HITS-CLIP (346 targets) was 4.5-fold higher (*P* < 0.001, binomial test) than that between our ADAR-only controls and HITS-CLIP (77 targets), indicating that the consistency between methods depends on Argonaute-guided editing and is not due to unspecific biases. Last, it is well established that Argonaute CLIP can identify miRNA binding events at high resolution, in some cases at the level of individual nucleotides^25^. We overlapped the editing positions following agoTRIBE transfection with reported eCLIP–seq binding sites, finding little consistency in the positional information conferred by the two methods—suggesting that our method might give less precise positional information than does eCLIP (Supplementary Fig. 3). Overall, our results show that agoTRIBE in its reported target repertoire resembles a CLIP-seq method even though it uses a completely distinct, antibody-free approach.

Besides experimental identification of miRNA–target interactions, computational methods that predict target sites are also available and commonly used^27–29^. These methods typically detect sequence motifs that could confer binding by specific miRNAs, and can also integrate their evolutionary conservation across species. Computational prediction inherently has high false-positive and -negative rates but, on the other hand, predicted miRNA binding sites that are conserved through evolution are likely functional bona fide miRNA target sites. In this way, we next compared our agoTRIBE top targets with transcripts predicted to be regulated by miRNAs according to the widely used TargetScan prediction database^28^, finding an overlap of 112 transcripts (Fig. 2h and Methods). This was threefold higher (*P* < 0.001) than the overlap with our ADAR-only control (37 transcripts), indicating that the convergence between agoTRIBE and the computational target prediction is due to Argonaute guidance. Additionally, we found that there was a 1.8-fold higher overlap (*P* < 0.001) between agoTRIBE and TargetScan predictions (112 transcripts) than between HITS-CLIP and TargetScan targets (62 transcripts). This provides sequence conservation evidence that agoTRIBE is more likely to report functional miRNA targets than this CLIP-seq method (Fig. 2i).

To investigate the functionality of agoTRIBE targets further, we performed global inhibition of miRNA action in human HEK-293T cells to detect derepressed targets. Because the TNRC6B protein is an essential cofactor in miRNA-driven, posttranscriptional repression, we performed inhibition of miRNA function by overexpressing the artificial T6B peptide, which effectively occupies the TNRC6B protein-binding pocket on Argonaute and causes global derepression of targets^30,31^ (Fig. 2j and Supplementary Fig. 4). We indeed observed a substantial derepression of TargetScan-predicted miRNA targets, suggesting that our perturbation experiment was successful (Fig. 2k and Supplementary Table 2). We next investigated the changes in gene expression of targets reported by agoTRIBE and HITS-CLIP and found that both target sets showed an 0.18 log_2_-fold increase in expression when miRNA function was inhibited (Fig. 2l and Supplementary Fig. 5). This became 0.20 and 0.22 log_2_-fold increases for the respective methods when only targets supported by sequence motif conservation were considered (Methods). These ~15% increases in expression are consistent with previous studies reporting ~30% derepression at the RNA and protein levels when miRNAs are strongly overexpressed or genetically deleted^32,33^. Furthermore, miRNAs have been proposed to have subtle functions in canalization of gene expression by buffering expression noise^34,35^. In summary, our experiment demonstrates that agoTRIBE predicts functional miRNA targets as efficiently as a state-of-the-art CLIP method.

The agoTRIBE approach tested here expresses an ADAR2 deaminase domain with Argonaute2 as a fusion protein from a single construct. To test the robustness of agoTRIBE we also benchmarked two distinct designs (Supplementary Fig. 6). The first relies on simultaneous expression and dimerization in living cells of an enhanced green fluorescent protein (eGFP)-tagged ADAR2 editing domain and a GFP nanobody fused with Argonaute2 (ref. ^36^). The second design consists of synthetic coiled-coil E3- and K3-tags, which enable heterodimer formation when Argonaute2 and the ADAR2 editing domain are individually coexpressed in living cells^37^. We found that both approaches increased endogenous editing relative to ADAR-only controls, demonstrating the robustness of agoTRIBE (Supplementary Fig. 7). In particular, these types of modular design give flexibility to experiments and allow an easy exchange of modifying domains (for example, TRIBE^12^, hyperTRIBE^13,14^ and STAMP^38,39^) and RNA-binding proteins of interest.

**Fig. 3:**
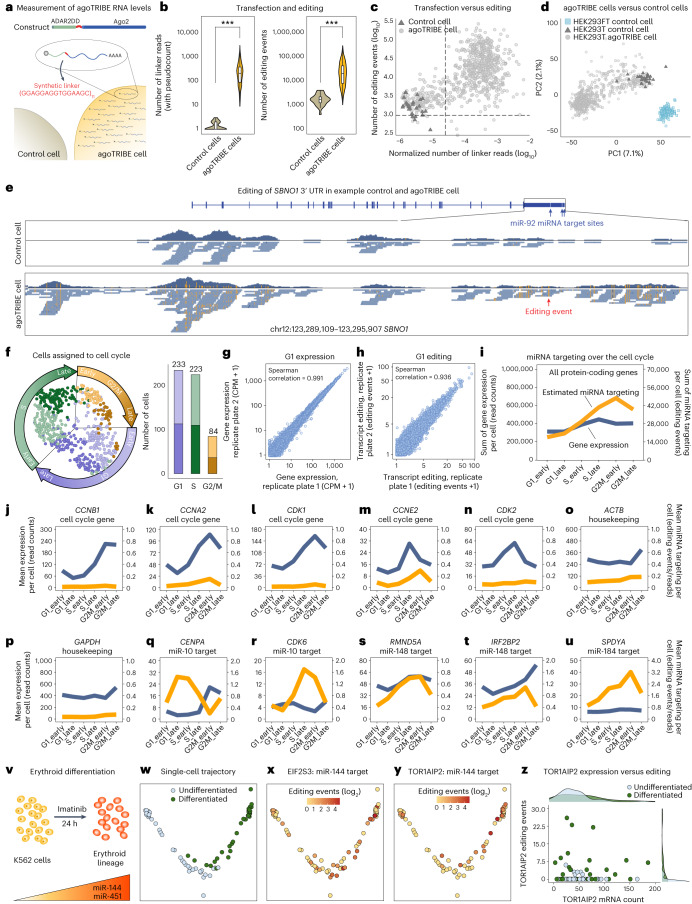
miRNA targeting in single cells. **a**, The agoTRIBE transcript includes an artificial linker that is detected in RNA-seq and can be used to estimate agoTRIBE levels. **b**, Levels of linker sequencing reads (left) and editing events (right) detected in 703 agoTRIBE-transfected cells and 26 control cells profiled by Smart-seq3 single-cell RNA-seq. Boxes indicate median and 25th and 75th quantiles, and whiskers indicate lowest and highest values. Significance was estimated using the Wilcoxon rank-sum test, with *P* < 0.0001. ***P < 0.0001. **c**, Normalized number of linker reads versus number of editing events for individual cells. The number of linker reads was normalized to the sequencing depth of each cell. Dashed lines indicate thresholds for agoTRIBE cells used for downstream analyses. **d**, Principal component (PC) analysis of agoTRIBE-transfected cells and control cells versus control cells from a different human embryonic kidney cell line (HEK-293FT). Cells were positioned based on their Smart-seq3 transcription profiles. **e**, Editing patterns of the *SBNO1* transcript in a control cell versus an agoTRIBE-transfected cell. **f**, Overview of 540 agoTRIBE-transfected single cells assigned to cell cycle stages using their Smart-seq3 profiles. Each cell cycle stage is divided into an early and late phase (Methods). Dimensionality reduction was performed with the UMAP algorithm. **g**, Gene expression for cells assigned to the G1 cell cycle stage. The cells originate from two distinct replicate plates of single cells. Each dot represents one gene. **h**, Transcript editing for cells assigned to the G1 cell cycle stage. **i**, Overview of estimated overall miRNA targeting during the cell cycle. Expression values and editing events were normalized to spike-ins and to the number of cells in each cell cycle stage (Methods). **j**–**u**, Examples of transcript expression and estimated miRNA targeting during the cell cycle: *CCNB1* (**j**), *CCNA2* (**k**), *CDK1* (**l**), *CCNE2* (**m**), *CDK2* (**n**), *ACTB* (**o**), *GAPDH* (**p**), *CENPA* (**q**), *CDK6* (**r**), *RMND5A* (**s**), *IRF2BP2* (**t**), *SPDYA* (**u**). Transcript expression is colored blue and miRNA targeting yellow. **v**, miR-144 and miR-451 increased in expression during K562 differentiation into erythroid precursor cells. **w**, Pseudotime trajectory of the differentiation process. Each dot indicates a single cell profiled by Smart-seq3. **x**,**y**, Editing in agoTRIBE-transfected cells during differentiation: EIF2S3 (**x**), TOR1AIP2 (**y**). **z**, Expression (read counts) and targeting (editing events) in TOR1AIP2. Each dot represents a single cell.

To test the sensitivity limits of our method we subjected agoTRIBE-transfected individual cells to Smart-seq3 single-cell RNA-seq^40^. In total, we profiled the transcriptomes of 703 agoTRIBE-transfected cells and 26 control HEK-293T cells following stringent quality controls (Methods and Supplementary Tables 5–7). Because the agoTRIBE construct includes an artificial linker region that is sequenced along with the transcriptomes, we could use sequence reads that map to the linker as an estimate of transfection efficiency of individual cells (Fig. 3a). As expected, many linker reads were detected in agoTRIBE-transfected cells while few linker reads—probably the result of mapping artifacts—were detected in control cells (Fig. 3b, left). Overall, transcriptome-wide editing increased substantially in agoTRIBE-transfected cells, with an average of ~32,500 editing events in each cell compared with ~1,900 in control cells (Fig. 3b, right). We also found that agoTRIBE cells with few linker reads tend to accumulate little editing (Fig. 3c), suggesting that some cells might not be efficiently transfected. However, these cells could easily be computationally identified and discarded, leaving a total of 540 efficiently transfected and edited cells for downstream analyses (Fig. 3c, dashed lines). We found that the transcriptional profiles of the agoTRIBE-transfected cells overall resemble those of control cells (Fig. 3d, light and dark gray, respectively). In contrast, control cells belonging to the fast-growing HEK-293FT cell line, but sequenced with the same protocol, clearly clustered separately from our control and agoTRIBE cells (Fig. 3d, light blue). These results suggest that editing by our fusion protein does not substantially alter transcriptome composition—even in measurements with sensitive single-cell methods such as the Smart-seq3 protocol.

We observed that specific miRNA targets increase strongly in editing in single cells. For instance, *SBNO1* has three binding sites for miR-92, which is a highly expressed miRNA in HEK-293T cells (Fig. 3e, blue arrows). The density plots of sequenced transcript parts show that the sensitive Smart-seq3 protocol yields transcript information for much of the 3′ untranslated region (UTR) in a control cell and an agoTRIBE-transfected cell (Fig. 3e, blue densities). Editing is virtually absent in the control cell but prevalent in the agoTRIBE cell (Fig. 3e, editing colored orange). Besides *SBNO1*, other high-confidence miRNA targets also have substantially increased editing in single cells transfected with agoTRIBE, showing the generality of our observations (Supplementary Fig. 8). As a proof of principle of the biological applications of our methods, we next applied Seurat^41^ to computationally sort the 540 agoTRIBE-transfected single cells into the G1, S and G2/M stages of the cell cycle (Fig. 3f). Even when stratifying cells into distinct cell cycle stages, our measurements of gene expression (Fig. 3g) and editing (Fig. 3h) were still highly reproducible. We found that overall normalized gene expression remained constant over the cell cycle while miRNA targeting events, as measured by global editing patterns, increased throughout the cell cycle from G1 to M (Fig. 3i). This is consistent with previous observations that miRNA repression is weakest in the G1 phase^42^, but the increase in editing could also represent accumulated miRNA targeting over the cell cycle that is then diluted by new transcription in the G1 phase^43,44^. While transcripts with longer half-lives tend to accumulate slightly more editing events than do shorter-lived transcripts (Supplementary Fig. 9), both groups display similarly increased editing throughout the cell cycle (Supplementary Fig. 10), lending some evidence to the former hypothesis that genuine increased miRNA repression is involved. We found that the agoTRIBE transcript is constant across the cell cycle while encoded protein decreases in G2/M, suggesting that our measurements of repression at this stage may in fact be underestimated (Supplementary Figs. 11 and 12). We found that known cell cycle-specific genes behaved as expected in our single-cell data (Fig. 3j–n). For instance, *CCNB1* has the highest expression in the G2/M stage, consistent with its role in promoting transition from G2 to the mitosis phase of the cell cycle (Fig. 3j). Similarly, *CDK2* is predominantly expressed in the S stage, consistent with its role in progression through the G1–S checkpoint (Fig. 3n). These specific cell cycle genes do not appear to be regulated by miRNAs, as evidenced by their low levels of editing across the entire cell cycle. In contrast, we found numerous genes differentially targeted by miRNAs across the cell cycle (Supplementary Table 8). For instance, the transcript of the centromeric protein CENPA appears to be strongly targeted by miRNAs during the G1 and S stages, but this targeting appears alleviated in G2/M where expression increased strongly, consistent with its role in mitosis (Fig. 3q). The *CDK6* gene has important roles in the G1–S transition and we found it to be specifically targeted in the S stage, where its expression might not be required (Fig. 3r). These examples serve as a proof of principle that agoTRIBE can detect miRNA targeting both in single cells and across distinct populations of single cells.

To test agoTRIBE on populations comprising distinct cell types we induced K562 cells to differentiate to erythroid precursor cells and applied Smart-seq3 to single cells (Supplementary Tables 9–11). It is well established that the cotranscribed miRNAs miR-144 and miR-451 are abundant and have important functions in erythrocytes^45^ (Fig. 3v). We found that both undifferentiated and differentiated cells separated well in a pseudotime trajectory, consistent with the linear and unidirectional nature of the differentiation (Fig. 3w). We found in total seven high-confidence targets of miR-144 that displayed increased targeting during this process. The protein TOR1AIP2 has functions in the endoplasmic reticulum and EIF2S3 is involved in translational initiation (Fig. 3x,y). Their repression during the differentiation process would be consistent with mature erythrocytes lacking an endoplasmic reticulum and having little or no translation. miRNA targeting appears to be heterogenous during this stage of erythroid differentiation, with cells either showing robust editing or no editing at all (Fig. 3x,y). We found that single cells that avoid targeting were still robustly detected in the sequencing (Fig. 3z), providing evidence that this heterogeneity in targeting is biological rather than technical in nature. In summary, we show that agoTRIBE can be applied to mixed-cell populations to give insights into heterogeneity in miRNA targeting.

## Discussion

In summary, here we present a method for detection of miRNA–target interactions transcriptome-wide in single cells. In a comparison with current state-of-the-art Argonaute CLIP-seq methods, we found that agoTRIBE has several advantages (Table 1). First, agoTRIBE does not require the use of antibodies but rather simple transfection, thus reducing the cost and time of the required experimental procedures by several days. Second, our method uses either ordinary bulk or single-cell RNA-seq to detect editing events, meaning that transcriptome-wide measurements of RNA levels are also provided as part of the protocol. Indeed, agoTRIBE transfection is so straightforward that it could be applied to any given standard RNA-seq experiment to provide transcriptome-wide miRNA–target interaction information at little additional cost or effort. Third, while Argonaute CLIP-seq requires millions of cells, agoTRIBE can be applied to individual cells.

**Table 1:** Comparison between Argonaute CLIP-seq methods and agoTRIBE

Method	Argonaute CLIP-seq	agoTRIBE
Starting input	Millions of cells	Down to single cells
Approach	Immunoprecipitation in lysed cells	Editing in living cells
Antibodies	Needed	Not needed
Specialized laboratory equipment	Needed	Not needed
Duration of protocol (excluding cell work)	>4 days	1.5–2.0 days
Exact miRNA binding positions	Available	Not available
Extra information	−	Provides RNA-seq data

Conversely, our agoTRIBE method is currently limited in the resolution with which it detects individual target sites, making it more useful as a method for detection of miRNA target transcripts rather than specific binding sites. This is in many ways similar to the first CLIP-based methods, which also often identified binding fragments hundreds of nucleotides long; this limitation may be alleviated by improved fusion protein design.

AgoTRIBE will allow us to study the heterogeneity of miRNA targeting in homogenous cell populations, about which little is currently known. It will also allow us to study miRNA targeting in complex cell compositions in cell culture—for instance, in organoids or during induced cell differentiation. The agoTRIBE fusion protein could, with some effort, be placed under an inducible promoter in a living organism, which would allow the profiling of miRNA targeting in individual cell types of a complex tissue such as mouse brain. Because editing events would be detected with single-cell RNA-seq, individual cells could be sorted into cell types using computational approaches and would not require any physical sorting. In conclusion, we foresee that agoTRIBE has numerous applications that will benefit the wider community and that it will facilitate the entry of the miRNA field into the single-cell era.

## Methods

### Ago2-tagging DNA construct and plasmids

DNA-encoding ADAR2DD_E488Q (human ADAR2 adenosine deaminase domain 316–701-amino acid (aa) hyperactive mutant E488Q, the ADAR-only control) and ADAR2DD_E488Q in-frame with 55-aa flexible linker and Ago2 (agoTRIBE) were chemically synthetized and inserted in pcDNA3.1(+) vector (GeneArt, Invitrogen/Thermofisher). The synthetic coiled-coil (EIAALEK)3, E3- and (KIAALKE)3, K3-tags were described previously^37^. The E3-GGSG linker-Ago2 and K3-GGSG linker-ADAR2DD_E488Q constructs were synthetized and inserted in pcDNA3.1(+) vector (GeneArt, Invitrogen/Thermofisher). Plasmids encoding EGFP-Ago2 and FLAG/HA-TNRC6B protein were described previously^46,47^. eGFP_ADAR2DD_E488Q was synthetized and cloned in pcDNA3.1(+) vector (GeneArt, Invitrogen/Thermofisher). The plasmid encoding GFP nanobody, pGNb-mCherry^36^ was used as pGNb-vector backbone. GNb-Ago2 and GNb-ADAR2DD_E488Q constructs were made by PCR from the corresponding complementary DNA, followed by insertion into the pGNb-vector lacking mCherry using Gibson assembly cloning (NEB). The following primers were used: Ago2-forward (GGGGGATCTGGATCCATGTACTCGGGAGCCGGCCCCGC);

Ago2-reverse (GCCCTCTAGACTCGAGTCAAGCAAAGTACATGGTGCGC);

ADAR2DD-forward (GGGGGATCTGGATCCATGCAGCTGCATTTACCGCAGG);

ADAR2DD–reverse (GCCCTCTAGACTCGAGTCAGGGCGTGAGTGAGAAC).

The DNA-encoding human T6B peptide (TRNC6B 599–683 aa^30^) and T6B fusion with GSG linker and mCherry were synthetized and cloned in pcDNA3.1(+) vector (GeneArt, Invitrogen/Thermofisher). The main constructs have been submitted to the Addgene repository with ID nos. 205598 (pcDNA3.1_ADAR2DD_E488Q), 205599 (pcDNA3.1_ADAR2DD_E488Q_Ago2) and 205600 (pcDNA3.1_T6B-mCherry).

### Cell culture and transfection

HEK-293T cells were grown in DMEM medium (Sigma) supplemented with 10% FBS (ThermoFisher) in the presence of penicillin/streptomycin (Sigma) under standard conditions at +37 °C in 5% CO_2_. Before transfection, cells were seeded in 12-well plates (Corning) at 60–70% confluency. Cells were transfected with 0.5–2.0 μg of the recombinant plasmid DNA in two biological replicates 18–24 h after seeding using Lipofectamine 3000 (Invitrogen) according to the manufacturer’s instructions.

### Immunofluorescence staining and microscopy

HEK-293T cells were seeded on four-well glass slides (Nunc Lab-TekII) at 60–70% confluency and transfected with 0.5–1.0 μg of the recombinant plasmid DNA using Lipofectamine 3000 (Invitrogen). The 48-h posttransfected cells were fixed with 3.7% formaldehyde (Sigma), permeabilized with PBS Tween containing 0.1% Triton X-100 (Sigma) and blocked in PBS (Gibco) with 1% bovine serum albumin (Sigma) and 0.1% Tween 20 (VWR). The primary antibodies against Ago2 and ADAR2 were added for 1 h at room temperature. After washing, secondary antibodies were added for 1 h in the dark. Cells were mounted using Vectashield mounting medium (Vector Laboratories) with 0.1 μg ml^–1^ DAPI (ThermoScientific) for DNA counterstaining. Cells were imaged with a ZOE fluorescent cell imager (Bio-Rad). The following antibodies were used: rabbit polyclonal human ADAR2 (no. GTX54916, GeneTex), mouse monoclonal Ago2 (no. ab57113, abcam), mouse monoclonal anti-Flag (no. F3165, Sigma), goat anti-rabbit IgG (H + L) Alexa 594 (no. A11012, ThermoFisher) and donkey anti-mouse IgG (H + L) Alexa Fluor Plus 488 (no. A32766, ThermoFisher).

### Bulk and single-cell RNA-seq

For standard bulk RNA-seq, cells were harvested 24 and 48 h after transfection. For bulk RNA library preparation, total RNA was isolated using the Quick-RNA miniprep kit (Zymo Research). Quality and integrity of RNA samples were assessed using a Bioanalyzer instrument and the RNA 6000 Nano kit (Agilent Technologies). One microgram of total RNA was used for RNA library preparation with the TruSeq Stranded mRNA kit (Illumina). Sequencing was carried out on an Illumina NextSeq 500. For single-cell RNA library preparation using the Smart-seq3 protocol, cells were harvested 48 h after transfection, stained with 1 μg ml^–1^ propidium iodide (Invitrogen) and sorted into 384-well plates using a BD Bioscience FACS Aria fusion instrument. Single-cell Smart-seq3 libraries were prepared at the Eukaryotic Single-Cell Genomics facility and sequenced on an Illumina NovaSeq 6000 platform at the NGI facility (SciLifeLab) as previously described^40^.

### Bulk and single-cell RNA-seq data preprocessing and mapping

Both bulk and single-cell RNA-seq data were first processed for quality and adapter trimming using cutadapt v.4.0, allowing a minimum length of 36 base pairs (bp) after trimming (with parameters *-*q 30 -m 36), and then mapped to the hg38 reference genome using STAR v.2.7.2b (ref. ^48^) with parameters detailed on GitHub (see link below). Putative PCR duplicates were removed using Picard using default options. The six samples used for estimation of transcriptome-wide editing levels (Supplementary Table 1) were subsampled to a sequencing depth of 18 million mapped reads using the Sambamba tool^49^ with the following command line options: view -f bam -t 20–subsampling-seed=3. We used the featureCounts tool^50^ for quantification of the expression level of genes based on Ensembl Hg38 genome annotation (https://www.ensembl.org/info/data/ftp/index.html, release v.99). Multimapping reads and/or reads overlapping by more than one feature (genes in our case) were accounted for using a fractional assignment (-M -O–fraction).

### Bulk and single-cell RNA editing analysis

Alignment mismatches were called using a custom variant-calling pipeline (available on GitHub: https://github.com/vaishnoviS/agoTRIBE.git) with two main filtering steps: (1) Phred scale-based quality filtering and (2) read depth-based filtering. All mismatches supported by Phred score >30 and at least two reads supporting the nucleotide substitutions were reported. Single-nucleotide substitutions were then intersected with positions in the single-nucleotide polymorphism (SNP) database (NCBI dbSNP v.151)^51^, and all known SNPs were excluded from further analyses. The remaining A-to-G and T-to-C (on reverse strand) substitutions were further compared with editing events compiled in the REDIportal database^52^ and known editing sites were excluded. Strand-aware A-to-G substitutions were then reported as editing events. For the purpose of identification of agoTRIBE-specific targets, the number of editing events was computed for each annotated 3′ UTR in protein-coding genes. Nonsense-mediated decay, nonstop decay, processed transcripts, retained introns and unprocessed pseudogene isoforms were removed. Regression analysis was performed with the editing events in agoTRIBE-transfected samples compared with ADAR-only control samples to identify 3′ UTRs having higher editing. Standard residual values were calculated based on linear regression applied on editing events per gene using the lm function from the R stats package. The top 1,000 3′ UTRs with highest residual values were considered as highly edited 3′ UTRs by agoTRIBE. Conversely, the 1,000 3′ UTRs with lowest residual values were considered as background ADAR-only targets.

### Small RNA-seq

Total RNA was isolated using the Quick-RNA miniprep kit (no. R1054, Zymo Research). The quality and integrity of RNA samples were assessed using a Bioanalyzer instrument and the RNA 6000 Nano kit (no. 5067-1511, Agilent Technologies). Small RNA libraries were prepared using the NextFlex small RNA library v.3 kit (no. 5132, BioScientific, Perkin Elmer Applied Genomics). The following amounts of total RNA were used as input: 0.25–1.00 μg of imatinib-treated K562 samples, 0.5 μg of agoTRIBE-transfected and untransfected HEK-293T control and 0.1 μg of cell cycle-specific populations (G0/G1, S, G2/M), and both agoTRIBE-transfected and untransfected HEK-293T control.

### Small RNA-seq analysis

Bulk small RNA-seq data were analyzed using miRTrace to trim adapter sequences. Processed reads from miRTrace were then quantified using quantify.pl from the miRDeep2 package. To identify differentially expressed miRNAs in control and agoTRIBE-transfected HEK-293T samples (Supplementary Fig. 2), DESeq2 was used on raw counts for miRNA and all miRNAs with *P*_adj._ < 0.01 were considered significant. For cell cycle analysis (Supplementary Fig. 13), reads per million (RPM) values obtained from quantify.pl were used to identify the top ten miRNAs in each stage and were plotted as a line plot.

### Overlaps between miRNA targets reported by agoTRIBE, CLIP-seq methods and TargetScan

Those genes with 3′ UTRs that were specifically edited in agoTRIBE samples (above) were compared with the top target genes from publicly available HITS-CLIP^25^, PAR-CLIP^24^ and eCLIP^26^ data. HITS-CLIP (Ago2 CLIP-Seq) and PAR-CLIP (AGO1234 PAR-CLIP) binding information was downloaded in BED format from the Dorina database^53^ and eCLIP data (GSE140367, GSM4247216, empty vector control rep 1 (AGO-eCLIP-seq)) were downloaded from NCBI GEO^54^. Targeted genes from HITS-CLIP and PAR-CLIP data were sorted based on total peak coverage, while genes from the eCLIP target was sorted based on *P* values reported in the GEO supplementary file. For each set, the top 1,000 genes were considered for further comparison with our agoTRIBE top 1,000 target genes. For the purpose of identifying top TargetScan targets, the ten most highly expressed miRNAs in HEK-293T cells were identified (miR-10a-5p, miR-92a-3p, miR-10b-5p, miR-191-5p, miR-148a-3p, miR-16-5p, miR-378a-3p, miR-182-5p, miR-222-3p and miR-186-5p, in descending order by expression) using our miRNA-seq data (Supplementary Table 13). The top 100 targets for each of these miRNAs (based on the cumulative context ++ score) were downloaded from the TargetScan database^28^. Overall, 699 unique high-confidence target genes were compiled for the top ten most highly expressed miRNAs in HEK-293T cells (given that some targets were shared between miRNAs and were redundant).

### Identification of target derepression following T6B transfection

Gene expression was estimated as described above. Unexpressed genes and those with <50 counts following normalization in any of the considered samples were removed. Between-sample normalization of expression was performed using the trimmed mean of the *M*-values method^55^. Fold change values between control and T6B-mCherry samples were calculated with the foldchange function from the gtools R package and visualized with their empirical cumulative distribution function. The top 699 TargetScan targets were defined as above, while the remaining expressed genes were defined as background.

### Identification of positional information in target editing

To identify whether agoTRIBE editing reflects miRNA binding positional information, we compared agoTRIBE editing positions with those reported by AGO-eCLIP–seq data (GSM4247216). To do so we focused on genes with monoexonic 3′ UTRs and their largest isoforms that showed one eCLIP editing event. Genes with 3′ UTRs shorter than 40 bp were discarded. We then calculated the distance (in bp) between agoTRIBE and eCLIP editing events if detected in the same gene. As background control we defined a random iteration of uniform distributions (*n* = 100) to generate dummy editing positions within each targeted 3′ UTR. Background distances were then computed using eCLIP positional data and randomly distributed editing sites.

### Single-cell RNA-seq cell filtering

Smart-seq3 data were preprocessed and mapped as described above. As a preliminary quality control step we filtered out cells with <9,000 detected genes or <150,000 deduplicated reads. To assess the efficiency of transfection we mapped our reads to the linker sequence with bowtie2 v.2.3.5.1 (ref. ^56^) using local alignment (*-*local) while allowing for one mismatch (–*N* 1), and quantified the aligned reads with the featureCounts v.2.0.0 tool^50^. For HEK-293T cells we then calculated normalized linker counts per cell as raw linker counts divided by the total number of reads, and plotted these against the editing events in 3′ UTRs. A pseudocount of 1 was added to all linker values to avoid division by zero. By visual inspection of the resulting plot we decided to exclude 163 cells with log_10_-normalized linker below −4.5 and below 1,000 editing events, leaving 566 cells in total. For K562 differentiated and undifferentiated cells, individual cells with a minimum of one linker count were considered positively transfected and used for further analysis. The K562 single-cell trajectory was constructed using transcriptomics counts with Monocle v.2.28.0 in R, with the top 1,000 genes differentially expressed between stages.

### Cell cycle assignment for single cells

Cell cycle annotation was based on the Seurat v.4.0 pipeline^41^. Cells were count per million normalized using the NormalizeData function from Seurat, with the normalization method of relative counts (normalization.method = ‘RC’) and a scale factor of 10^6^ (scale.factor = 1,000,000). We then annotated cells into cell cycle phases. To do this, we customized the CellCycleScoring function from Seurat to better fit Smart-seq3 data and used it with the default markers provided by Seurat. Specifically, cells with *S*-score and G2/M-score (as defined by the Seurat software) >50 were assigned to G1. These same marker genes were considered for scaling of data and calculation of the first 50 principal components with the Seurat functions ScaleData and RunPCA and default parameters. Finally, uniform manifold approximation and projection (UMAP) dimensionality reduction on the first ten dimensions was performed with the RunUMAP function. Based on this graphical representation, every cell cycle phase was split into ‘early’ and ‘late’ substages, with both substages containing approximately the same number of cells. For the purpose of identifying changes in gene expression and miRNA targeting across the cell cycle, sequence data from all cells belonging to a given cell cycle substage were pooled and mapped to Thermofisher ERCC92 spike-in sequences using bowtie, with strict parameters (-v 1) and a pseudobulk approach. The number of reads mapping to the spike-in was divided by the corresponding number of cells in the specific subphase to derive a scaling factor. This scaling factor was then used to normalize expression values of the pooled cells mapping to each cell cycle substage.

### Transcript half-life analyses

The top 20% of stable and unstable transcripts was obtained from ref. ^57^ (GEO: GSE49831). For the violin plot (Supplementary Fig. 9), the per-transcript sum of expression and sum editing over all agoTRIBE-transfected cells (540) were calculated. For cell phase analysis (Supplementary Fig. 10), cells from each cell cycle phase were pooled and expression and editing were calculated. Normalization was performed as in Fig. 3i.

### Erythroid differentiation from K562 cells

K562 cells (1.0–1.3 × 10^5^ ml^–1^) were cultured in 12-well plates containing RPMI (no. 21875034, Gibco) with 10% fetal bovine serum and penicillin/streptomycin supplemented with 5 mM imatinib (no. 9084 S, Cell Signaling) for erythroid differentiation. Cells cultured in RPMI supplemented with 10% FBS, penicillin/streptomycin and 5 mM DMSO (no. 8418, Sigma) were used as an undifferentiated control. The Neon transfection system (no. MPK 1096, Invitrogen) and 1 μg of agoTRIBE and ADAR-only constructs were used for K562 electroporation. Cells harvested 24 h after electroporation and corresponding DMSO (no. D8418, Sigma) controls were used for Smart-seq3 preparation.

### Cell sorting by cell cycle stage

An asynchronous population of un- and transfected HEK-293T cells was used for cell cycle analysis and cell sampling for small RNA-seq. HEK-293T cells were transfected with 2.5 μg of agoTRIBE- and ADAR-only constructs using Lipofectamine 3000 and harvested 48 h after transfection. Following cell cycle analysis and cell sorting, cells were stained with Vybrant DyeCycle Green (no. V35004, Invitrogen) and propidium iodide. A BD Bioscience FACS Aria fusion cytometer was used for cell sorting in the G1, S and G2/M phases as determined by Vybrant DyeCycle Green staining.

### Cell cycle profiling of agoTRIBE protein levels

HEK-293T cells were transfected with 2.5 μg of the agoTRIBE recombinant plasmid DNA using Lipofectamine 3000, then 48-h posttransfected cells were harvested. The suspended cells were washed with PBS (no. 10010-031, Gibco), fixed with 3.7% formaldehyde (no. 47608, Sigma), permeabilized with PBS Tween containing 0.1% Triton X-100 (no. T8787, Sigma) and blocked in PBS with 10% FBS, 0.36 μM IgG (no. 026202, Life Technologies) and 0.1% Tween 20 (no. 0777, VWR). The primary antibody against ADAR2 (no. GTX54916, GeneTex) was added for 30 min at room temperature and the secondary antibody, goat anti-rabbit IgG Alexa 594 (no. A11012, ThermoFisher), was added for 20 min in the dark. After washing with PBS, cells were resuspended in 400 μl of PBS and stained with Vybrant DyeCycle Green and 30 nM DAPI (no. 62248, ThermoScientific). A negative control with IgG and without ADAR2 primary antibody was used for Alexa 594 compensation, and the agoTRIBE Alexa 594-positive cell population was used for cell cycle analysis. G0/G1, S and G2/M phases were determined using Vybrant DyeCycle Green staining and a BD Bioscience FACS Aria fusion cytometer. agoTRIBE protein expression was determined as mean Alexa 594 fluorescence intensities normalized to G0/G1, S and G2/M event counts.

### Statistical analyses

Differences in fold change derepression following T6B transfection in the top 1,000 edited genes by agoTRIBE and in miRNA targets according to HITS-CLIP, compared with background unedited and/or nontargeted genes, were tested with the DTS test, which calculates a reweighted integral of the distance between two empirical cumulative distributions, available in the dts_test function from the twosamples R package. Overlap significance between agoTRIBE targets, CLIP targets and TargetScan targets was estimated using binomial statistics. Differences in long linker reads and in editing events comparing control samples and samples following transfection with agoTRIBE were assessed with a nonparametric approach using the Wilcoxon rank-sum test.

#### AI protein structure predictions

The human Ago2 (Q9UKV8) three-dimensional protein structure was predicted by AlphaFold2 DB v.1 (ref. ^19^). K3/E3 peptides, eGFP and GNb and anti-GFP nanobody (LaG-16) structure predictions were generated using the Robetta, RoseTTAFold^18^ modeling method available at https://robetta.bakerlab.org/.

### Reporting summary

Further information on research design is available in the Nature Portfolio Reporting Summary linked to this article.

## Online content

Any methods, additional references, Nature Portfolio reporting summaries, source data, extended data, supplementary information, acknowledgements, peer review information; details of author contributions and competing interests; and statements of data and code availability are available at 10.1038/s41587-023-01951-0.

### Supplementary information

Supplementary Information Supplementary Figs. 1–13.

Reporting Summary

Supplementary Table 1 Overview of all bulk datasets.

Supplementary Table 2 Expression value for all genes for six samples in Fig. 1.

Supplementary Table 3 Editing positions in 3′ UTR for 6 samples in Fig. 1.

Supplementary Table 4 Editing events in 3′ UTR for six samples in Fig. 1.

Supplementary Table 5 Overview of HEK-293T single cells. Cell ID, agoTRIBE or control, Seurat-inferred cell state, reads mapping to Linker sequence.

Supplementary Table 6 Expression value for all genes for quality control-processed HEK-293T single cells.

Supplementary Table 7 Editing events in 3′ UTR for quality control-processed HEK-293T single cells.

Supplementary Table 8 Differentially edited genes between cell cycle stages for HEK-293T cells.

Supplementary Table 9 Overview of K562 single cells. Cell ID, differentiated or undifferentiated, reads mapping to Linker sequence.

Supplementary Table 10 Expression value for all genes for K562 single cells.

Supplementary Table 11 Editing events in 3′ UTR for K562 single cells.

Supplementary Table 12 Overview of all bulk small RNA-seq datasets.

Supplementary Table 13 Expression values for all miRNAs for all bulk small RNA-seq datasets.

## Data Availability

Sequencing data have been deposited at SRA under accession no. PRJNA994505 (ref. ^58^).

## Code availability

The source used in this study is available at GitHub: https://github.com/vaishnoviS/agoTRIBE (ref. ^59^).
