# Supplementary material for: Detection of transcriptome-wide microRNA–target interactions in single cells with agoTRIBE
Source: Nat Biotechnol. 2023 Sep 21;42(8):1296–302. doi: 10.1038/s41587-023-01951-0 (PMC11324520; doi:10.1038/s41587-023-01951-0)
Supplement: Supplementary file 1 — Supplementary Figs. 1–13. [file 41587_2023_1951_MOESM1_ESM.pdf]

nature biotechnology

Article

https://doi.org/10.1038/s41587-023-01951-0

Detection of transcriptome-wide microRNA–target interactions in single cells with agoTRIBE

In the format provided by the authors and unedited

Table of Contents

Supplemental Figures **2**

Figure S1 *2*

Figure S2 *3*

Figure S3 *4*

Figure S4 *5*

Figure S5 *6*

Figure S6 *7*

Figure S7 *8*

Figure S8 *9*

Figure S9 *13*

Figure S10 *14*

Figure S11 *15*

Figure S12 *16*

Figure S13 *17*

Supplemental Tables **18**

Table1 *18*

Table2 *18*

Table3 *18*

Table4 *18*

Table5 *18*

Table6 *18*

Table7 *18*

Table8 *18*

Table9 *18*

Table10 *18*

Table11 *18*

Table12 *18*

Table13 *18*

Supplemental Figures

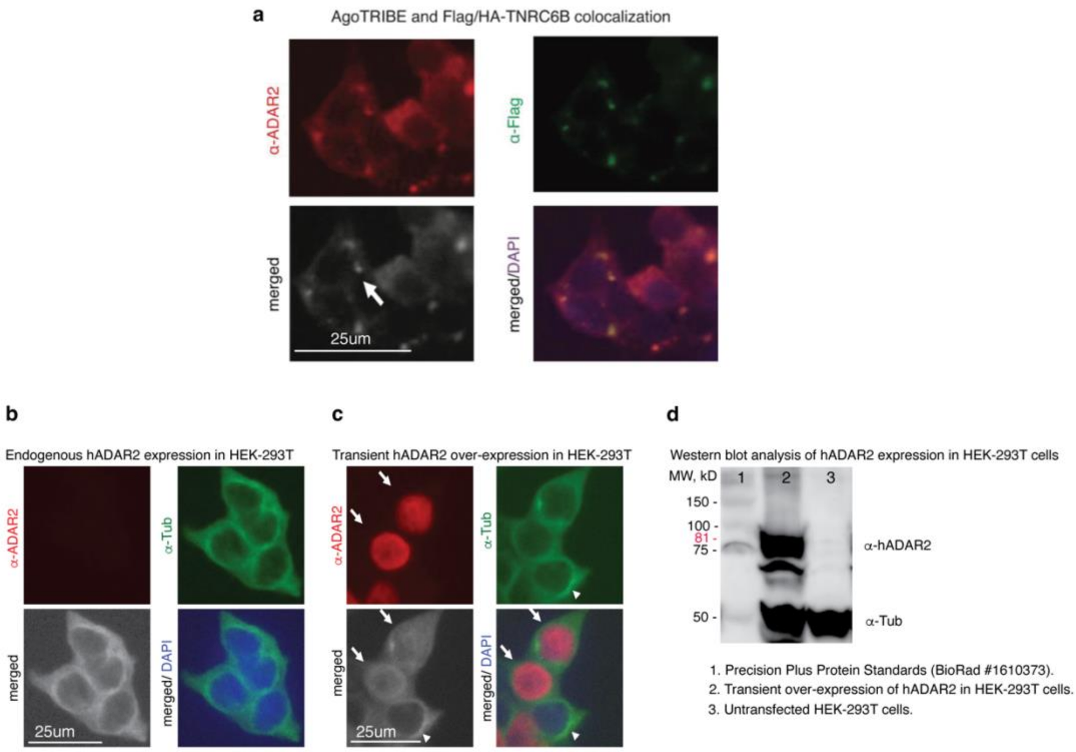

Figure S1. agoTRIBE and Flag/HA-TNRC6B colocalization and lack of detection of endogenous ADAR2 in HEK293T cells. **a,** Transiently co-expressed agoTRIBE and Flag/HA-TNRC6B are colocalized in the cytoplasm with P-bodies. Anti-ADAR2 detects agoTRIBE (red), the P-bodies are labelled by Flag/HA-TNRC6B (green), a merged image with DAPI. **b**, Endogenous ADAR2 expression is not detectable in immunolabeled HEK-293T cells. **c**, Human ADAR2 staining is nuclear (nucleolar) in HEK-293T cells transiently overexpressing human full length ADAR2 WT protein (shown by arrows). The arrowhead shows a neighbouring un-transfected HEK-293T cell lacking ADAR2 staining. **d**, Western blotting analysis of endogenous ADAR2 expression in HEK-293T cells. 40μg of the whole cell extracts of HEK-293T cells transfected with a plasmid expressing hADAR2 WT and un-transfected cells control were separated on SDS-PAGE and simultaneously blotted with polyclonal rabbit α-ADAR2 (GenTex GTX54916) and monoclonal anti-α-Tubulin (Sigma T5168). The antibodies were simultaneously developed with goat anti-rabbit IgG-HRP (Abcam ab6721) and goat anti-mouse IgG, HRP conjugated (Sigma SAB3701066), and Immobilon Forte Western HRP substrate (Millipore, Merck WBLUF0500). The 81kD and 50kD bands corresponding to hADAR2 and loading control Tubulin are indicated (lane 2 and lanes 2-3, respectively).

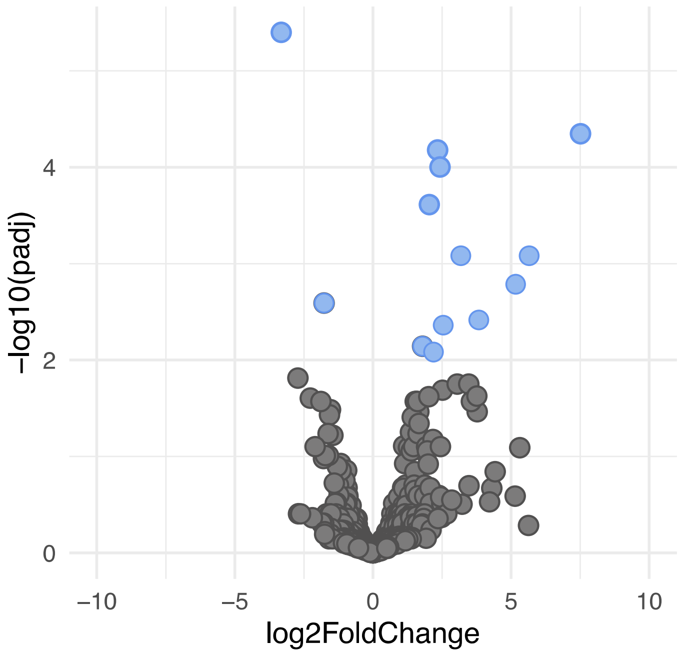

Figure S2: miRNA expression change upon agoTRIBE transfection. In total 3 batches of HEK293T cells transfected with agoTRIBE construct and 3 batches of control cells were subjected to small RNA-seq (Methods). DESeq2 identified 13 miRNAs that were differentially expressed in agoTRIBE-transfected cells, using the Benjamini-Hochberg procedure (two-sided). However, these were all lowly abundant, with none of the 13 miRNAs ranking among the top 50 expressed miRNAs in either agoTRIBE or control cells according to the small RNA-seq data.

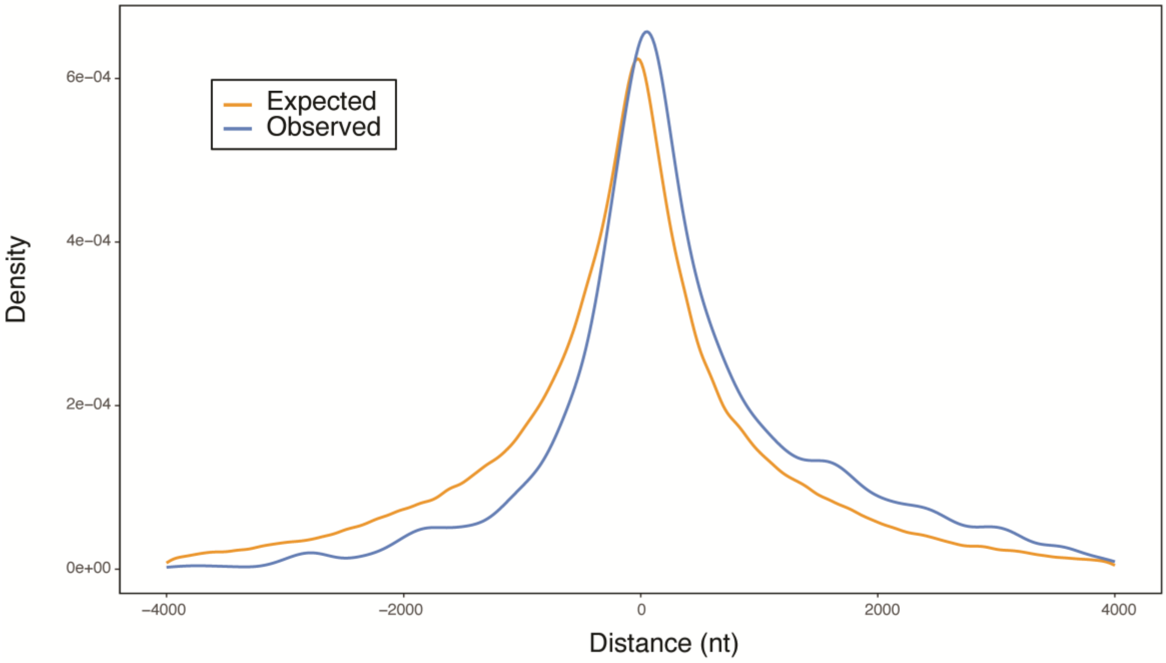

Figure S3. Positional information in target editing. The observed distance (in nt) between the experimentally reported eCLIP binding site and the editing positions identified by agoTRIBE in the longest mono-exonic 3’ UTRs per gene. Expected distance is the result of randomizing editing positions following a uniform distribution across the 3' UTRs and comparing them with eCLIP reported binding sites.

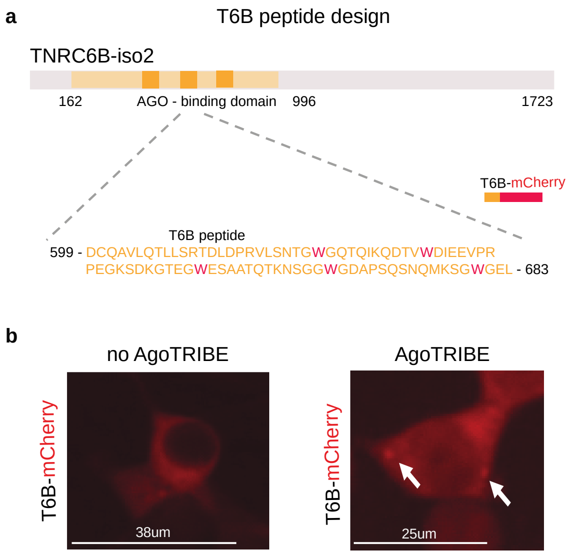

Figure S4. T6B peptide design. a, Schematic representation of TNRC6B Ago-binding domains and T6B, the short TNRC6B-derived protein peptide fused with mCherry. **b**, Transient expression of T6B-mCherry and agoTRIBE in HEK293T cells. In presence of agoTRIBE, T6B-mCherry is localized in cytoplasmic foci similar to P-bodies; highlighted with the arrows. All microscopy experiments were performed in at least two replicates and a representative experiment shown.

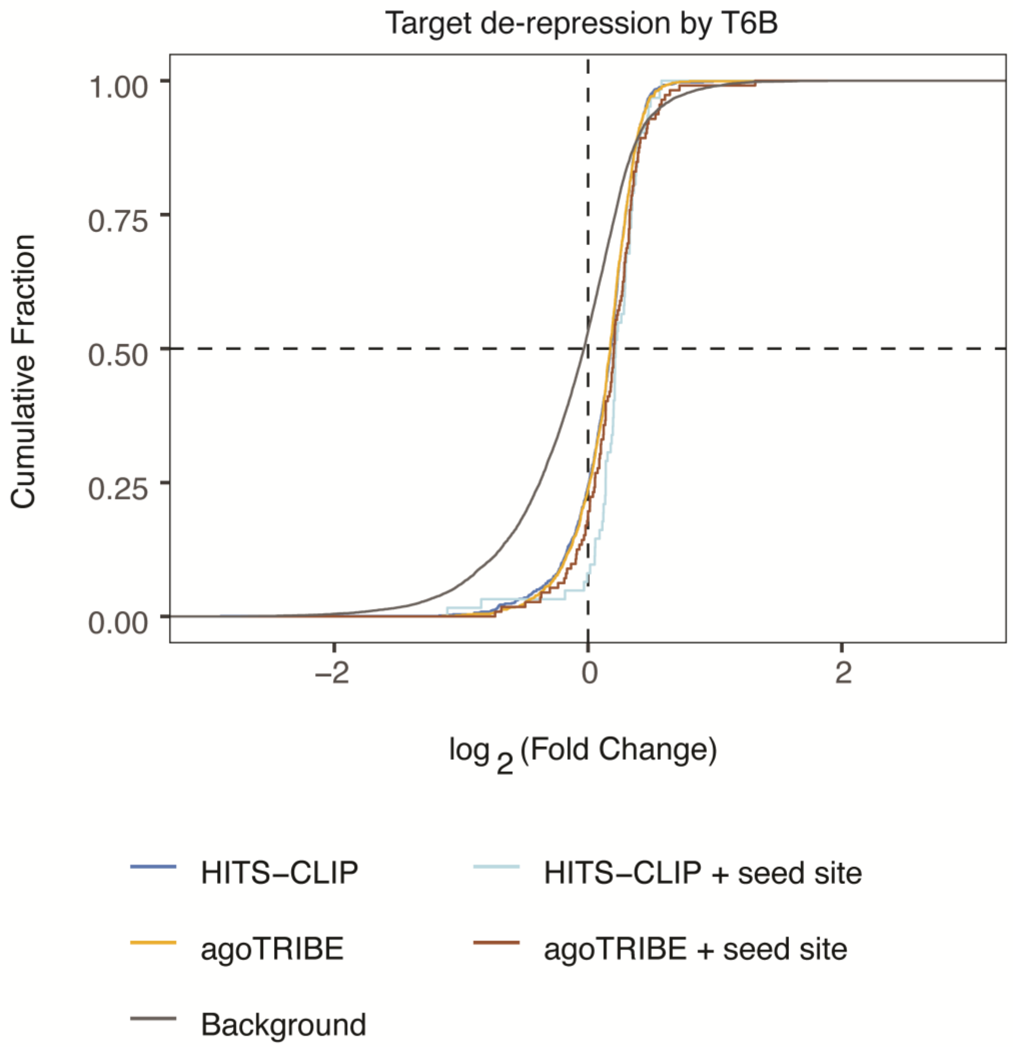

Figure S5. De-repression of miRNA targets identified by agoTRIBE and HITS-CLIP upon T6B-mCherry transfection. The targets identified by AgoTRIBE and HITS-CLIP are excluded from 'Background’. ‘agoTRIBE+seed site’ consists of the targets identified by agoTRIBE which are high confidence TargetScan targets of at least one of the 10 most abundant miRNA in HEK-293T cells. Similarly, ‘HITS-CLIP+seed site’ consists of the targets identified by HITS-CLIP which are high confidence TargetScan targets of at least one of the 10 most abundant miRNA in HEK-293T cells.

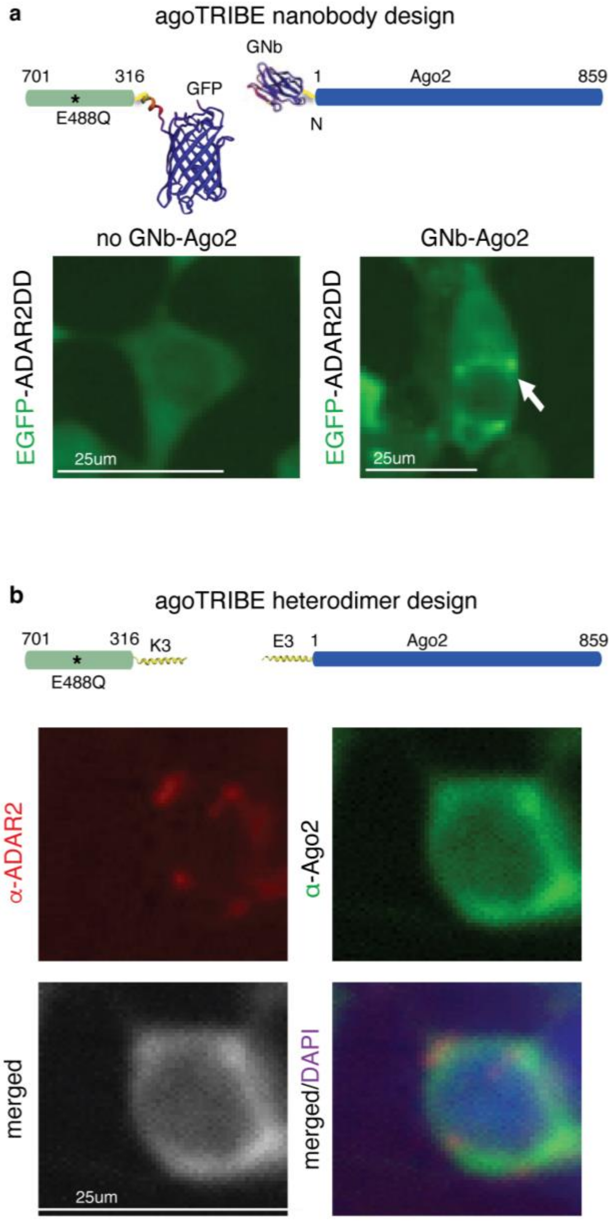

Figure S6. Modular agoTRIBE. a, Nanobody fusion allows specific interaction with an EGFP-fused ADAR2DD in the cytosol of live cells. HEK-293T cells transfected with constructs transiently expressing GFP-nanobody fused with Ago2 (GNb_Ago2) and EGFP-tagged ADAR2DD. In presence of GNb_Ago2, EGFP-ADAR2DD segregates into cytoplasmic foci similar to P-bodies, highlighted with an arrow. **b**, Inducible heterodimerization of Ago2 and ADAR2DD by E3 and K3, the synthetic coiled-coil tags. Transient co-expression and localization of E3_Ago2 with K3_ADAR2DD, immunolabeled with anti-ADAR2 (red) and anti-Ago2 (green). **a**-**b** merged images with DAPI. All microscopy experiments were performed in at least two replicates and a representative experiment shown.

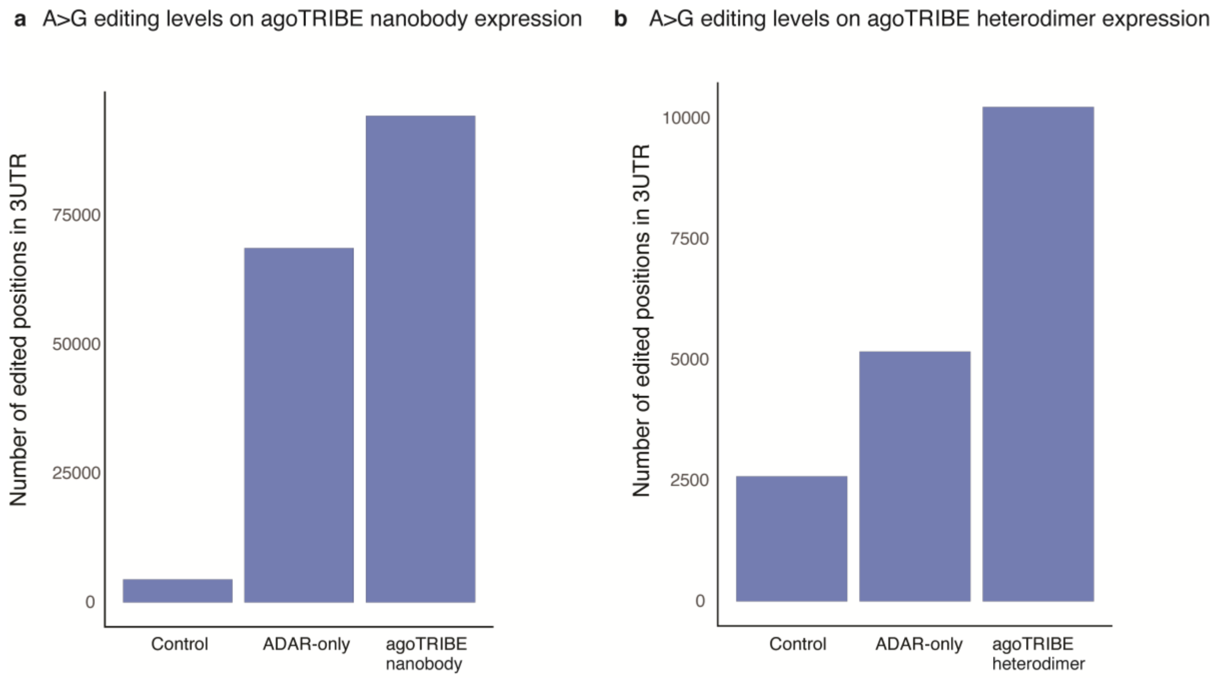

Figure S7. A>G editing in 3' UTRs for agoTRIBE nanobody and heterodimer designs. a, A>G editing indicative of ADAR2 editing increases in agoTRIBE (eGFP-ADAR2DD and GNb-Ago2) transfected cells compared to control cells (GNb-Ago2) and cells transfected with the ADAR2 editing domain without Argonaute2 (eGPF-ADARDD: 'ADAR-only' controls), however the ADAR-only control in nanobody design gives a relatively higher background. **b,** A>G editing indicative of ADAR2 editing increases in agoTRIBE (K3-ADAR2DD and E3-Ago2) transfected cells compared to control cells (E3-Ago2) and cells transfected with the ADAR2 editing domain without Argonaute2 (K3-ADARDD: 'ADAR-only' controls).

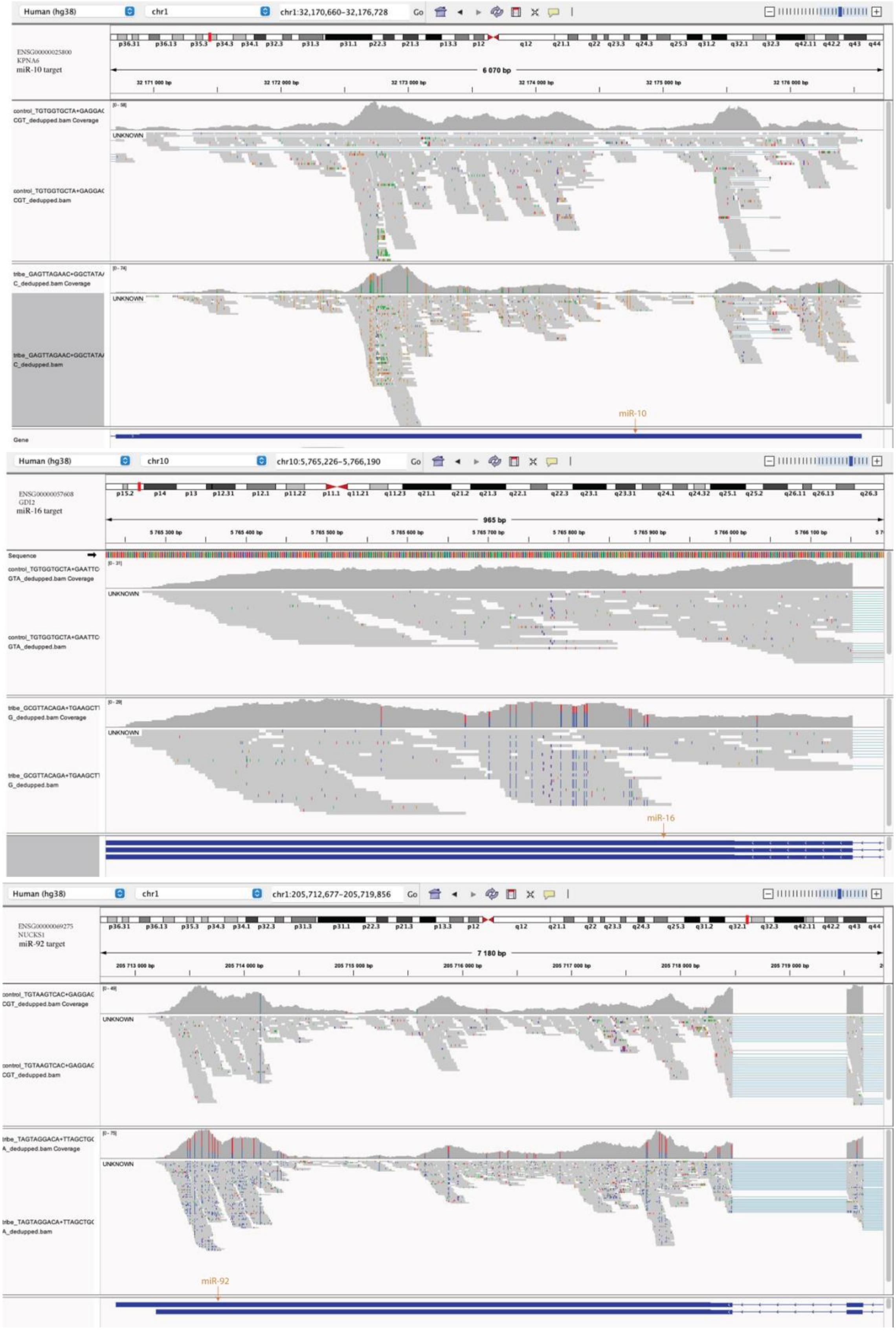
Human (hg38)
chr1
chr1:32,170,660-32,176,728
ENSG00000025800
KPNA6
miR-10 target
6 070 bp
control_TGTGGTGCTA+GAGGAC
CGT_dedupped.bam Coverage
control_TGTGGTGCTA+GAGGAC
CGT_dedupped.bam
tribe_GAGTTAGAAC+GGCTATA
C_dedupped.bam Coverage
tribe_GAGTTAGAAC+GGCTATA
C_dedupped.bam
UNKNOWN
miR-10
Gene
Human (hg38)
chr10
chr10:5,765,226-5,766,190
ENSG00000057608
GDI2
miR-16 target
965 bp
Sequence
control_TGTGGTGCTA+GAATTC
GTA_dedupped.bam Coverage
control_TGTGGTGCTA+GAATTC
GTA_dedupped.bam
tribe_GCGTTACAGA+TGAAGCT
G_dedupped.bam Coverage
tribe_GCGTTACAGA+TGAAGCT
G_dedupped.bam
miR-16
Human (hg38)
chr1
chr1:205,712,677-205,719,856
ENSG00000069275
NUCKS1
miR-92 target
7 180 bp
control_TGTAAGTCAC+GAGGAC
CGT_dedupped.bam Coverage
control_TGTAAGTCAC+GAGGAC
CGT_dedupped.bam
tribe_TAGTAGGACA+TTAGCTGC
A_dedupped.bam Coverage
tribe_TAGTAGGACA+TTAGCTGC
A_dedupped.bam
miR-92

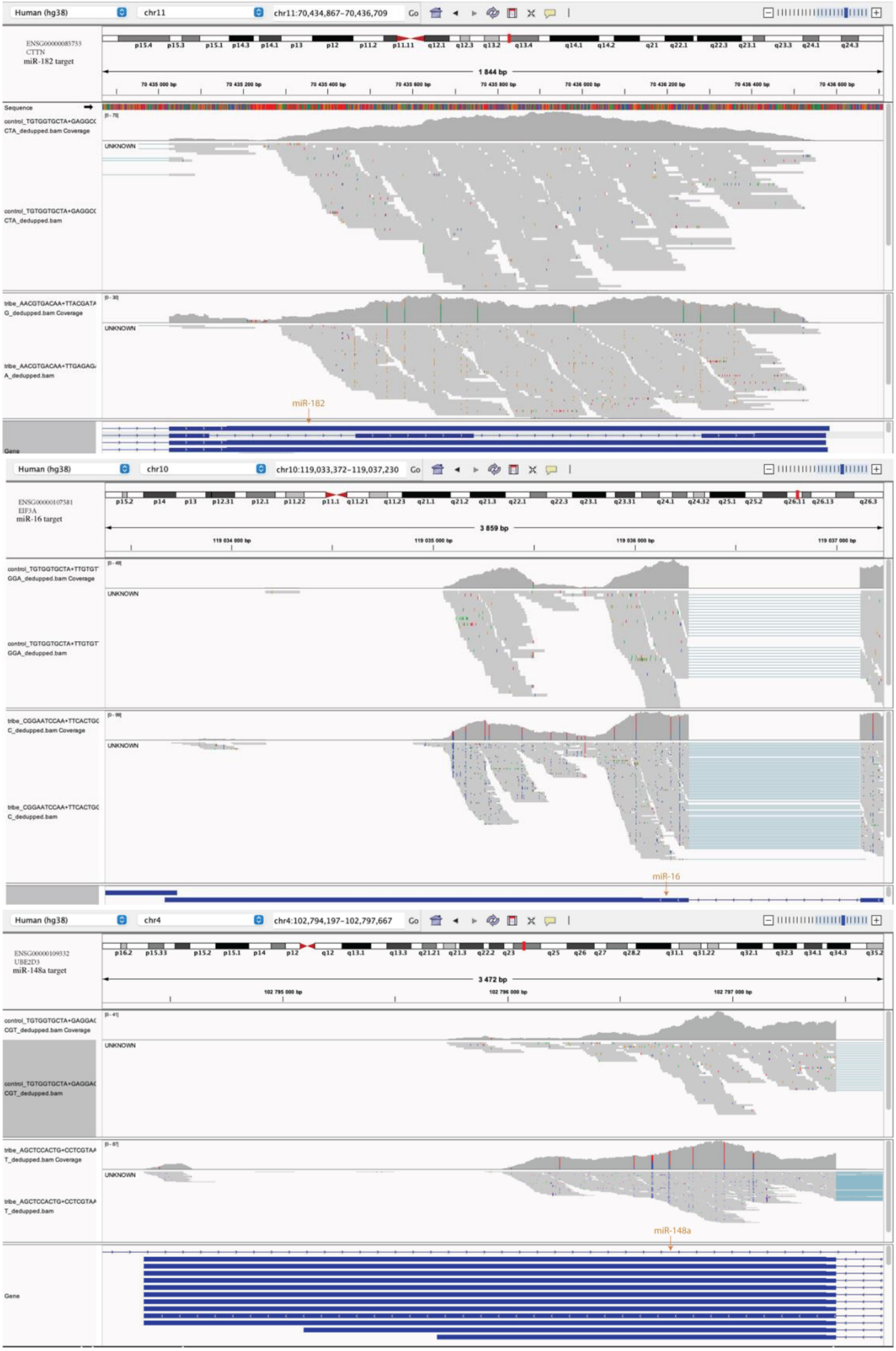
Human (hg38)
chr11
chr11:70,434,867-70,436,709
ENSG00000085733
CTTN
miR-182 target
1 844 bp
Sequence
UNKNOWN
miR-182
Gene
Human (hg38)
chr10
chr10:119,033,372-119,037,230
ENSG00000107581
EIF3A
miR-16 target
3 859 bp
UNKNOWN
miR-16
Human (hg38)
chr4
chr4:102,794,197-102,797,667
ENSG00000109332
UBE2D3
miR-148a target
3 472 bp
UNKNOWN
miR-148a
Gene

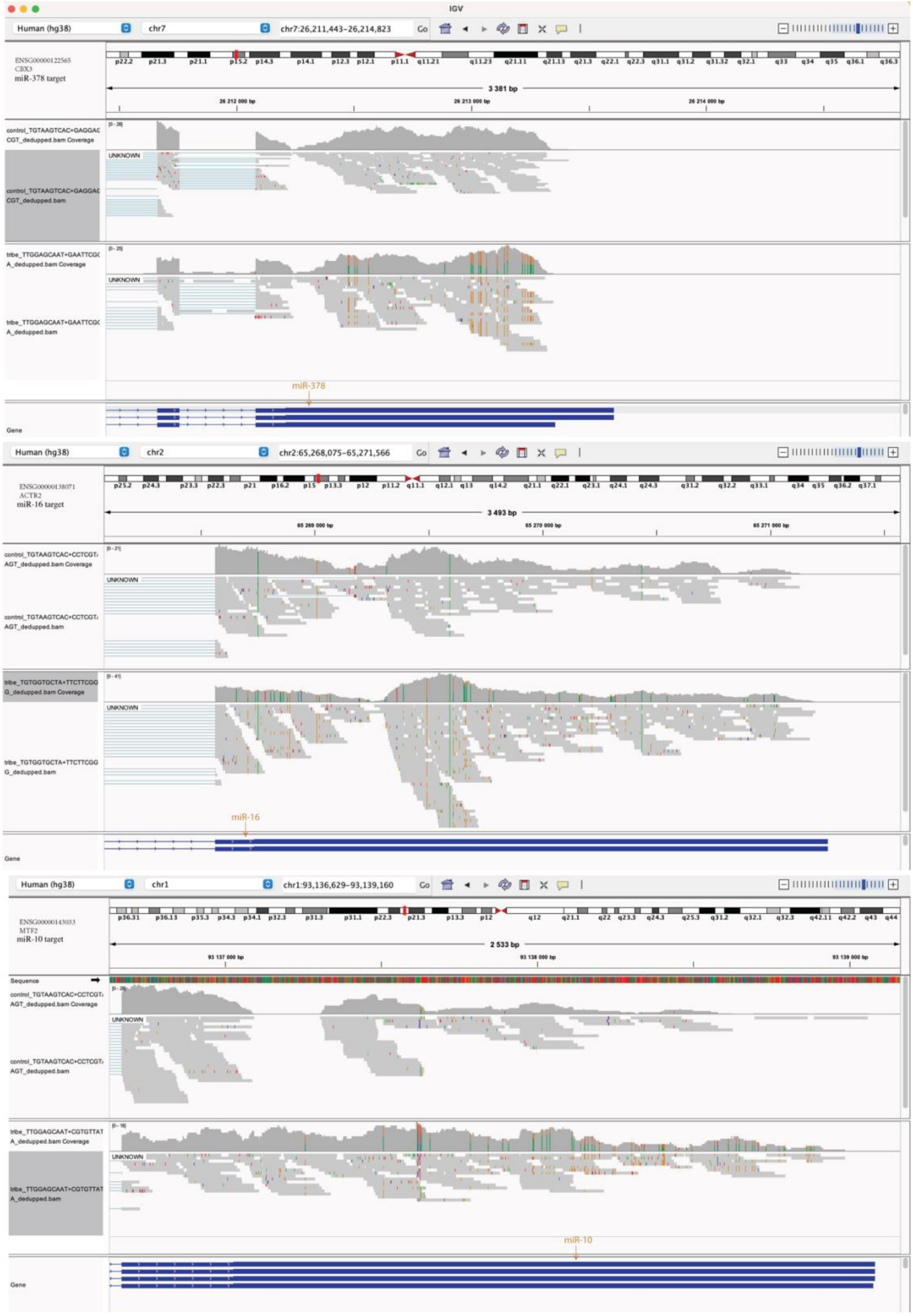
IGV
Human (hg38)
chr7
chr7:26,211,443-26,214,823
ENSG00000122565
CBX3
miR-378 target
3 381 bp
control_TGTAAGTCAC+GAGGACGT_dedupped.bam Coverage
control_TGTAAGTCAC+GAGGACGT_dedupped.bam
tribe_TTGGAGCAAT+GAATTCGCA_dedupped.bam Coverage
tribe_TTGGAGCAAT+GAATTCGCA_dedupped.bam
UNKNOWN
miR-378
Gene
Human (hg38)
chr2
chr2:65,268,075-65,271,566
ENSG00000138071
ACTR2
miR-16 target
3 493 bp
control_TGTAAGTCAC+CCTCGTAGT_dedupped.bam Coverage
control_TGTAAGTCAC+CCTCGTAGT_dedupped.bam
tribe_TGTGGTGCTA+TTCTTCGGG_dedupped.bam Coverage
tribe_TGTGGTGCTA+TTCTTCGGG_dedupped.bam
miR-16
Gene
Human (hg38)
chr1
chr1:93,136,629-93,139,160
ENSG00000143033
MTF2
miR-10 target
2 533 bp
Sequence
control_TGTAAGTCAC+CCTCGTAGT_dedupped.bam Coverage
control_TGTAAGTCAC+CCTCGTAGT_dedupped.bam
tribe_TTGGAGCAAT+CGTGTTATA_dedupped.bam Coverage
tribe_TTGGAGCAAT+CGTGTTATA_dedupped.bam
miR-10
Gene

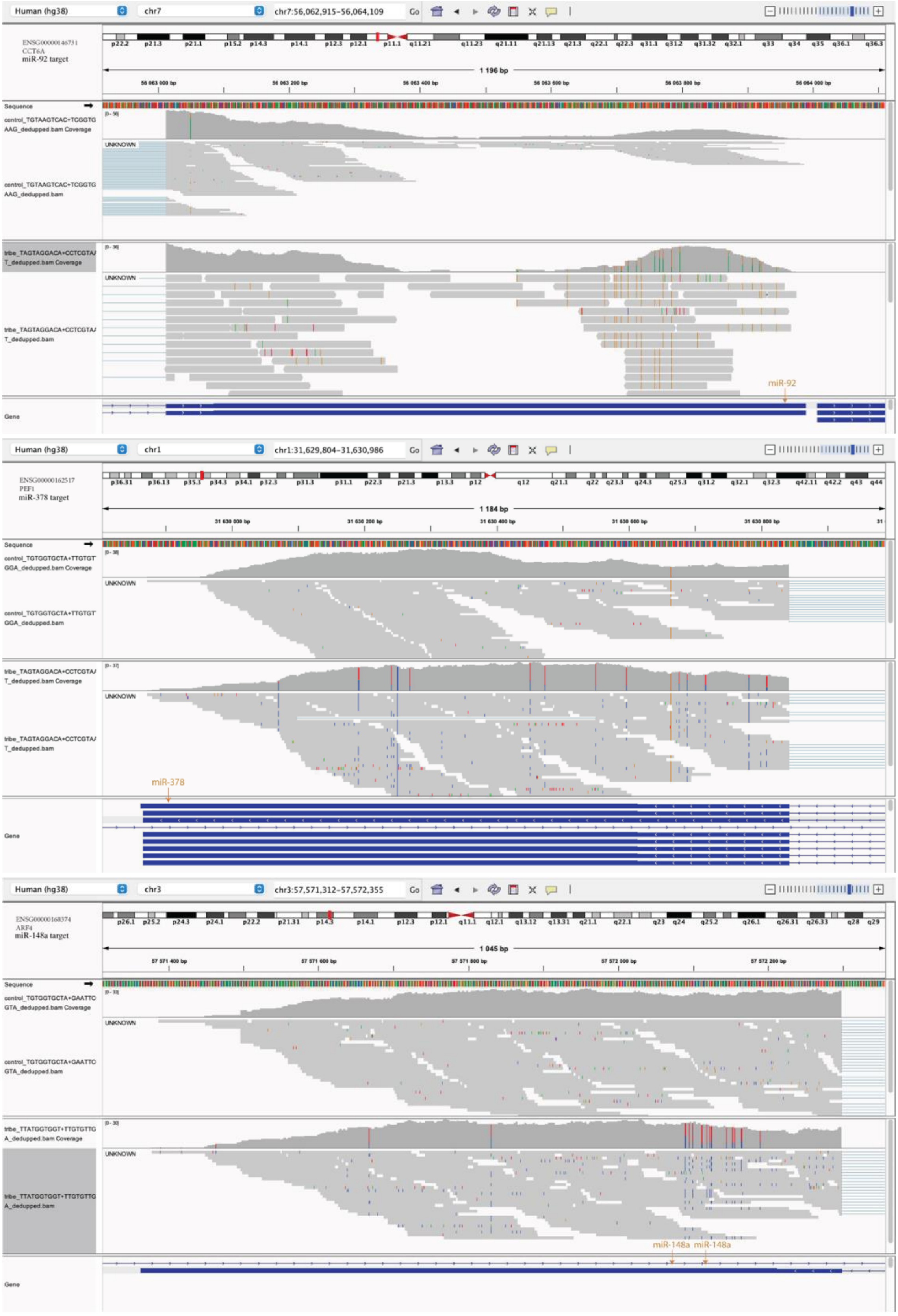

Figure S8. Editing patterns of the miRNA target transcripts. Each screenshot shows the editing pattern in a control cell vs. an agoTRIBE transfected cell for one miRNA targets (similar to Figure 3e). The miRNA binding site for most expressed miRNA is indicated with arrow and name of the miRNA.

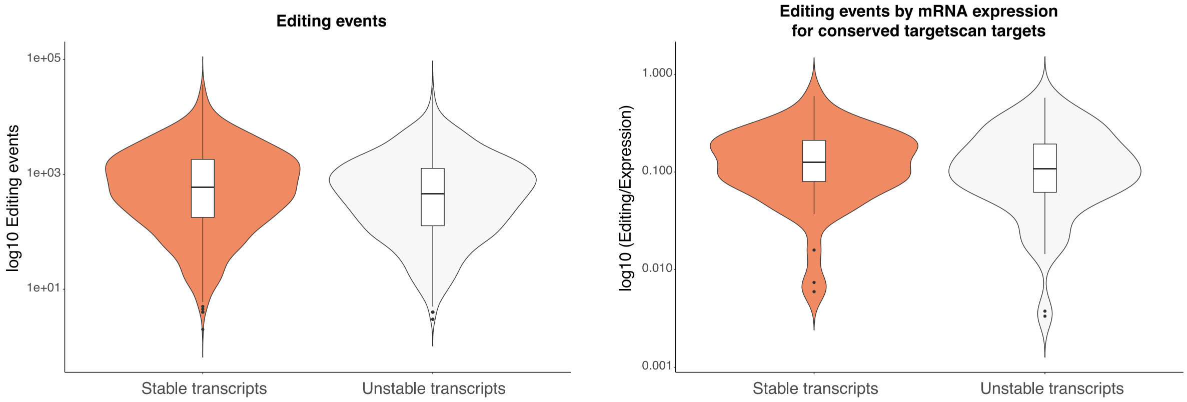

Figure S9: editing levels of stable and unstable transcripts. The 20% transcripts with the longest half-lives according to published data (Schueler, Munschauer et al., Genome Biology, 2014) were considered ‘stable transcripts’ and the 20% with the shortest half-lives were considered ‘unstable transcripts’. Boxes indicate median and 25th and 75th quantiles, and the whiskers indicate smallest and largest values. Overall, the stable transcripts had more editing events summed over the 540 HEK293T single cells (599 editing events) than did the unstable transcripts (455 editing events). When normalizing the number of editing events to expression levels (by dividing the number of editing events with the sum of expression read counts over the 540 HEK293T cells) and considering only transcripts that were high-confidence TargetScan targets of the top 10 most abundant miRNAs in this cell line, the stable transcripts still showed more editing (0.13 edits/read counts) than did the unstable transcripts (0.11 edits/read counts).

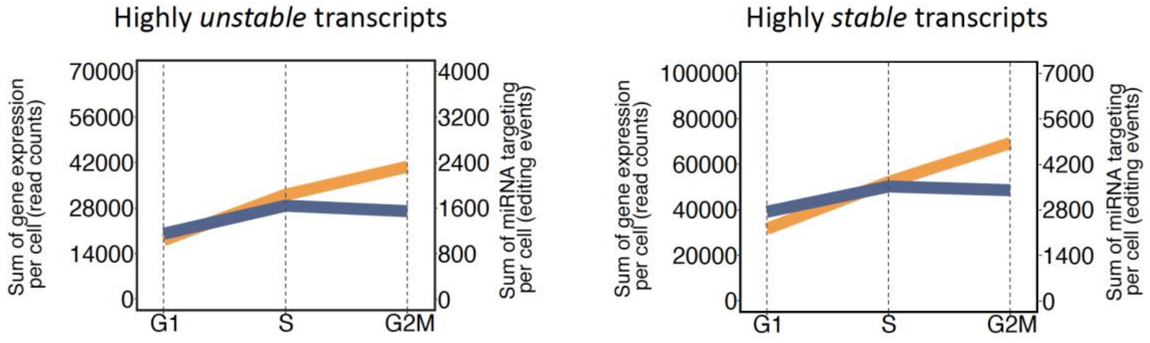

Figure S10: Expression and targeting of stable and unstable transcripts over the cell cycle. The estimated overall miRNA targeting (in yellow) during cell cycle for 20% transcripts with the longest half-lives ('stable transcript') and 20% transcript with the shortest half-lives ('unstable transcripts'). Expression values and editing events were normalized to spike-ins and to the number of cells in each cell cycle stage, similar to Figure 3i (Methods).

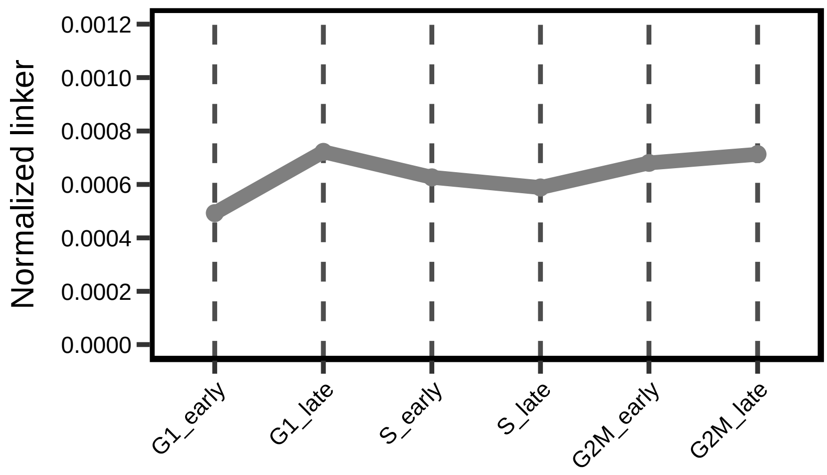

Figure S11: Expression of agoTRIBE transcript over the cell cycle. The expression is estimated by the linker reads that are normalized to mRNA read counts, similar to Figure 3c (Methods).

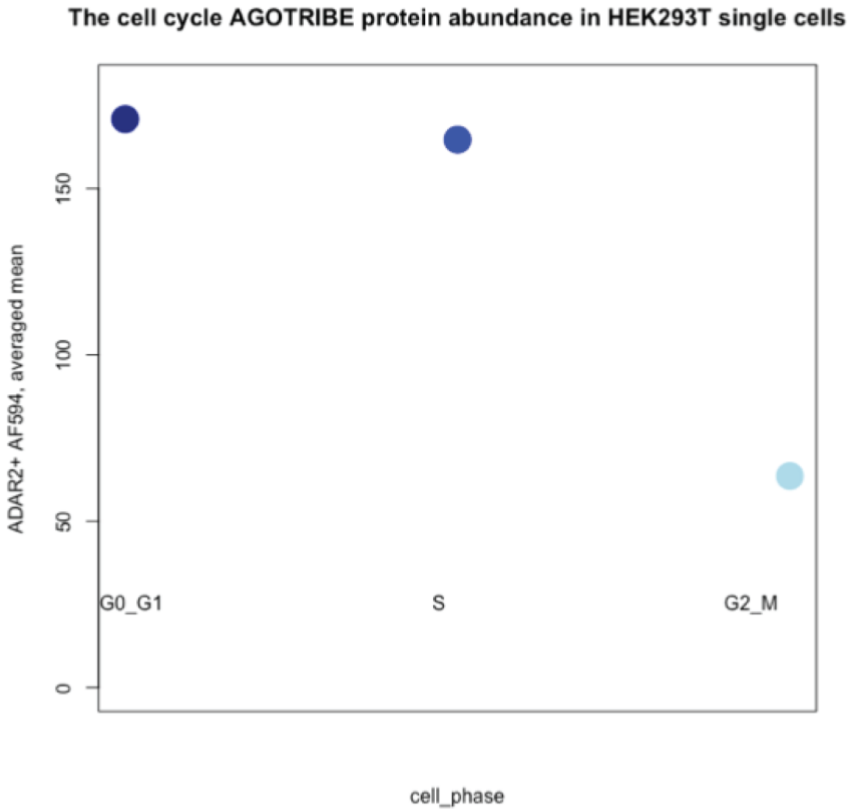

Figure S12: Expression of the AGOTRIBE protein over the cell cycle. Protein expression was profiled by fluorescently labeled anti-ADAR2 antibody (Methods).

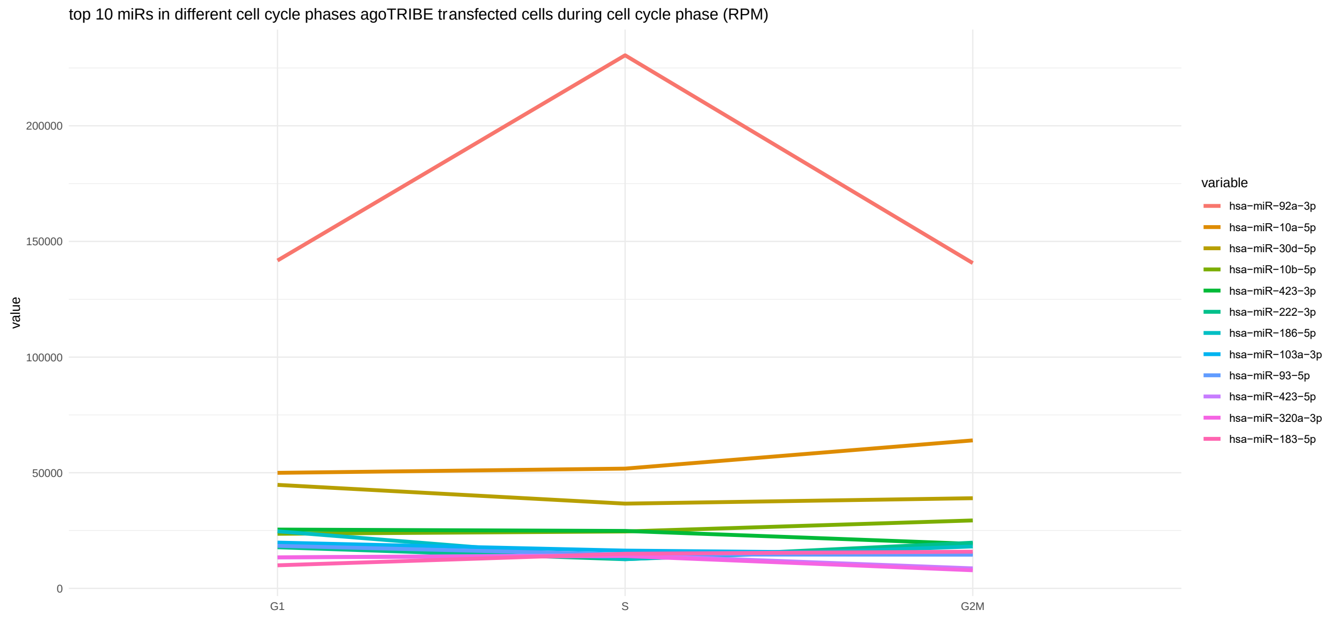

Figure S13: miRNA expression in agoTRIBE transfected cells across the cell cycle. 10 most highly expressed miRNAs in each cell cycle stages are shown. Expression is normalized to RPM (reads per million values, Methods).

Supplemental Tables

Supplementary Tables are available at
https://github.com/vaishnoviS/agoTRIBE/tree/main/SupplementaryTables
Table1: Overview of all the bulk datasets
Table2: Expression value for all genes for 6 samples in main figure 1
Table 3: Editing positions in 3' UTR for 6 samples in main figure 1
Table 4: Editing events in 3' UTR for 6 samples in main figure 1
Table 5: Overview of the HEK293T single cells - Cell ID, agoTRIBE or control. Seurat inferred cell state, reads mapping to Linker sequence
Table6: Expression value for all genes for quality control processed HEK293T single cells
Table7: Editing events in 3' UTR for quality control processed HEK293T single cells
Table8: Differentially edited genes between cell cycle stages in HEK293T cells
Table9: Overview of the K562 single cells – Cell ID, differentiated or undifferentiated, reads mapping to Linker sequence
Table10: Expression value for all genes for K562 single cells
Table11: Editing events in 3' UTR for K562 single cells
Table12: Overview of all the bulk small RNA-seq datasets
Table13: Expression value for all miRNA for all bulk small RNA-seq datasets
