# Supplementary material for: Detection of transcriptome-wide microRNA–target interactions in single cells with agoTRIBE
Source: Nat Biotechnol. 2023 Sep 21;42(8):1296–302. doi: 10.1038/s41587-023-01951-0 (PMC11324520; doi:10.1038/s41587-023-01951-0)
Supplement: Supplementary file 2 — Reporting Summary [file 41587_2023_1951_MOESM2_ESM.pdf]

nature portfolio

Corresponding author(s): Marc R. Friedländer, Inna Biryukova

Last updated by author(s): 8/8/2023

Reporting Summary

Nature Portfolio wishes to improve the reproducibility of the work that we publish. This form provides structure for consistency and transparency in reporting. For further information on Nature Portfolio policies, see our Editorial Policies and the Editorial Policy Checklist.

Statistics

For all statistical analyses, confirm that the following items are present in the figure legend, table legend, main text, or Methods section.

n/a	Confirmed	
☐	☒	The exact sample size (*n*) for each experimental group/condition, given as a discrete number and unit of measurement
☐	☒	A statement on whether measurements were taken from distinct samples or whether the same sample was measured repeatedly
☐	☒	The statistical test(s) used AND whether they are one- or two-sided<br>*Only common tests should be described solely by name; describe more complex techniques in the Methods section.*
☒	☐	A description of all covariates tested
☒	☐	A description of any assumptions or corrections, such as tests of normality and adjustment for multiple comparisons
☐	☒	A full description of the statistical parameters including central tendency (e.g. means) or other basic estimates (e.g. regression coefficient) AND variation (e.g. standard deviation) or associated estimates of uncertainty (e.g. confidence intervals)
☐	☒	For null hypothesis testing, the test statistic (e.g. *F*, *t*, *r*) with confidence intervals, effect sizes, degrees of freedom and *P* value noted<br>*Give P values as exact values whenever suitable.*
☒	☐	For Bayesian analysis, information on the choice of priors and Markov chain Monte Carlo settings
☒	☐	For hierarchical and complex designs, identification of the appropriate level for tests and full reporting of outcomes
☒	☐	Estimates of effect sizes (e.g. Cohen's *d*, Pearson's *r*), indicating how they were calculated

Our web collection on statistics for biologists contains articles on many of the points above.

Software and code

Policy information about availability of computer code

Data collection	No software was used for data collection.
Data analysis	cutadapt v. 4.0<br>STAR v. 2.7.2b<br>Sambamba tool v.0.7.1<br>featureCounts v2.0.0<br>bowtie2 v 2.3.5.153<br>Seurat v4.0<br>AlphaFold2 DB v1<br>Monocle v2.28.0<br>Various R packages as described in the methods section<br>Exact command lines used are available at: https://github.com/vaishnoviS/agoTRIBE

For manuscripts utilizing custom algorithms or software that are central to the research but not yet described in published literature, software must be made available to editors and reviewers. We strongly encourage code deposition in a community repository (e.g. GitHub). See the Nature Portfolio guidelines for submitting code & software for further information.

Data

Policy information about availability of data

All manuscripts must include a data availability statement. This statement should provide the following information, where applicable:

- Accession codes, unique identifiers, or web links for publicly available datasets
- A description of any restrictions on data availability
- For clinical datasets or third party data, please ensure that the statement adheres to our policy

Data availability
Sequencing data have been deposited at SRA under this accession number: PRJNA994505

Code availability
The source used in this study is available at GitHub: https://github.com/vaishnoviS/agoTRIBE

Public data and databases utilized in the study:
NCBI dbSNP v.151
REDIportal database
hg38 reference genome from NCBI
hg38 reference annotation from Ensembl database (https://www.ensembl.org/info/data/ftp/index.html, release version 99)
HITS-CLIP (Ago2 CLIP-Seq) and PAR-CLIP (AGO1234 PAR-CLIP) targets downloaded as BED format from Dorina database
eCLIP data (GSE140367, GSM4247216, Empty vector control rep 1 [AGO-eCLIP-seq]) was downloaded from NCBI GEO database
Predicted miRNA target information was retrieved from TargetScan Human database.

All of the intermediate data files with gene expression values, editing events etc are available as Supplementary Tables.

Human research participants

Policy information about studies involving human research participants and Sex and Gender in Research.

Reporting on sex and gender	No human research participants were included in this study.
Population characteristics	-
Recruitment	-
Ethics oversight	-

Note that full information on the approval of the study protocol must also be provided in the manuscript.

Field-specific reporting

Please select the one below that is the best fit for your research. If you are not sure, read the appropriate sections before making your selection.

☒ Life sciences ☐ Behavioural & social sciences ☐ Ecological, evolutionary & environmental sciences

For a reference copy of the document with all sections, see nature.com/documents/nr-reporting-summary-flat.pdf

Life sciences study design

All studies must disclose on these points even when the disclosure is negative.

Sample size	Sample size was chosen based on the single cell technology available. Smart-seq3 was run on two 384 well plates, covering control cells and agoTRIBE-transfected cells, for both HEK293T cells and K562 cells.
Data exclusions	Individual cells were excluded based on poor quality statistics, as described in the Methods section.
Replication	Reproducibility of agoTRIBE in single cells was tested by comparing gene expression and editing events in the distinct 384 well plates (Figure 3g-h).
Randomization	Samples were allocated into experimental groups based on the treatment, for instance control cells vs. agoTRIBE-transfected cells.
Blinding	We did not use blinding in our analyses, since the computational scientists were aware of the cell treatments before starting the analyses. Understanding the nature of the cell treatments is necessary for conducting the analyses.

Reporting for specific materials, systems and methods

We require information from authors about some types of materials, experimental systems and methods used in many studies. Here, indicate whether each material, system or method listed is relevant to your study. If you are not sure if a list item applies to your research, read the appropriate section before selecting a response.

Materials & experimental systems

n/a	Involved in the study
☐	☒ Antibodies
☐	☒ Eukaryotic cell lines
☒	☐ Palaeontology and archaeology
☒	☐ Animals and other organisms
☒	☐ Clinical data
☒	☐ Dual use research of concern

Methods

n/a	Involved in the study
☒	☐ ChIP-seq
☒	☐ Flow cytometry
☒	☐ MRI-based neuroimaging

Antibodies

Antibodies used

Rabbit polyclonal human ADAR2 (GeneTex, GTX54916), lot number 822003857 and 822102256.
Mouse monoclonal Ago2 (Abcam, ab57113), clone 2E12-1C9, lot number gr3186789-1.
Mouse monoclonal anti-Flag (Sigma, F3165), clone M2, lot number SLBL1237V.
Mouse monoclonal anti-α-Tubulin (Sigma, T5168), clone B-5-1-2, lot number 115M4828V.

Validation

GTX54916 validation by manufacturer https://www.genetex.com/Product/Detail/ADAR2-antibody/GTX54916 and in our study, Sekar et al, Supplementary Figure 1b-d and Figure 1c.
ab57113 validation by manufacturer https://www.abcam.com/products/primary-antibodies/argonaute-2-antibody-2e12-1c9-bsa-and-azide-free-ab57113.html
ab57113 has been referenced in 150 publications https://www.abcam.com/products/primary-antibodies/argonaute-2-antibody-2e12-1c9-bsa-and-azide-free-ab57113.html
F3165 validation by manufacturer http://www.sigmaaldrich.com/catalog/product/sigma/f3165?lang=en&region=SE, references are available from https://www.sigmaaldrich.com/SE/en/product/sigma/f3165.
T5168 enhanced validation by manufacturer https://www.sigmaaldrich.com/SE/en/product/sigma/t5168, references are available from https://www.sigmaaldrich.com/SE/en/product/sigma/t5168

Eukaryotic cell lines

Policy information about cell lines and Sex and Gender in Research

Cell line source(s)

Standard HEK-293T cells were used, obtained from the D. Kanellis/J. Bartek lab. Standard K562 (ATCC® CCL-243TM) were used, obtained from G. Baselli (Vicent Pelechano lab)

Authentication

HEK-293T cell line was authenticated using cell line authentication test by Eurofins Genomics. The K562 cell line was not authenticated.

Mycoplasma contamination

Both HEK-293T and K562 cells were specifically tested for Mycoplasma contamination.

Commonly misidentified lines
(See ICLAC register)

We have not worked with any commonly misidentified cell lines.
